# Drought-induced molecular changes in crown of various barley phytohormone mutants

**DOI:** 10.1080/15592324.2024.2371693

**Published:** 2024-06-26

**Authors:** Anetta Kuczyńska, Martyna Michałek, Piotr Ogrodowicz, Michał Kempa, Natalia Witaszak, Michał Dziurka, Damian Gruszka, Agata Daszkowska-Golec, Iwona Szarejko, Paweł Krajewski, Krzysztof Mikołajczak

**Affiliations:** a Institute of Plant Genetics, Polish Academy of Sciences, Poznań, Poland; b Faculty of Natural Sciences, The Franciszek Górski Institute of Plant Physiology Polish Academy of Sciences, Krakow, Poland; c Institute of Biology, Biotechnology and Environmental Protection, Faculty of Natural Sciences, University of Silesia in Katowice, Katowice, Poland

**Keywords:** Abiotic stress, brassinosteroids, functional annotation, gibberellins, mRNA sequencing, stress-induced proteins, strigolactones

## Abstract

One of the main signal transduction pathways that modulate plant growth and stress responses, including drought, is the action of phytohormones. Recent advances in omics approaches have facilitated the exploration of plant genomes. However, the molecular mechanisms underlying the response in the crown of barley, which plays an essential role in plant performance under stress conditions and regeneration after stress treatment, remain largely unclear. The objective of the present study was the elucidation of drought-induced molecular reactions in the crowns of different barley phytohormone mutants. We verified the hypothesis that defects of gibberellins, brassinosteroids, and strigolactones action affect the transcriptomic, proteomic, and hormonal response of barley crown to the transitory drought influencing plant development under stress. Moreover, we assumed that due to the strong connection between strigolactones and branching the *hvdwarf14.d* mutant, with dysfunctional receptor of strigolactones, manifests the most abundant alternations in crowns and phenotype under drought. Finally, we expected to identify components underlying the core response to drought which are independent of the genetic background. Large-scale analyses were conducted using gibberellins-biosynthesis, brassinosteroids-signaling, and strigolactones-signaling mutants, as well as reference genotypes. Detailed phenotypic evaluation was also conducted. The obtained results clearly demonstrated that hormonal disorders caused by mutations in the *HvGA20ox2*, *HvBRI1*, and *HvD14* genes affected the multifaceted reaction of crowns to drought, although the expression of these genes was not induced by stress. The study further detected not only genes and proteins that were involved in the drought response and reacted specifically in mutants compared to the reaction of reference genotypes and *vice versa*, but also the candidates that may underlie the genotype-universal stress response. Furthermore, candidate genes involved in phytohormonal interactions during the drought response were identified. We also found that the interplay between hormones, especially gibberellins and auxins, as well as strigolactones and cytokinins may be associated with the regulation of branching in crowns exposed to drought. Overall, the present study provides novel insights into the molecular drought-induced responses that occur in barley crowns.

## 1. Introduction

Plants are sessile organisms; therefore, they must adapt their growth and architecture to changing environments. Phytohormones are fundamental messengers that modulate plant responses to stress stimuli.^1^ Understanding how genes and hormones interact to coordinate plant growth under abiotic and biotic stress is a major challenge in developmental biology. Gibberellins (GAs), brassinosteroids (BRs), and strigolactones (SLs) are the principal groups of growth-promoting phytohormones in barley, and disorders in their biosynthesis or signaling pathways may lead to perturbations in plant development and affect plant tolerance to stress, including drought.^2^

GA homeostasis is primarily regulated by GA 20-oxidases (GA20ox), 3-oxidases (GA3ox), and 2-oxidases (GA2ox).^3^ GA20ox and GA3ox catalyze the oxidation of C-20 and C-3 in GA molecules, respectively, thereby activating GAs. In contrast, GA2ox is a crucial enzyme for the deactivation of GAs. The loss of function of *GA20ox* or *GA3ox* decreases the level of GA and leads to a reduction in plant height, whereas overexpression stimulates growth. Increased expression of *GA2ox* causes dwarfism by decreasing internode elongation.^4,5^ Both GA-sensitive and GA-insensitive barley mutants exist,^6,7^ and mutations in the *HvGA20ox2* gene (*sdw1*), which encodes GA-20 oxidase, have been identified.^4,8^ Generally, reduced levels of GA in plants confer improved drought resistance (reviewed by;^9^ for instance, the overexpression of *GA2ox* enhances the drought tolerance of rice.^10^ In contrast, the exogenous application of GA to GA-sensitive transgenic tomatoes resulted in decreased drought resistance compared with non-treated tomato mutants.^11^

Brassinosteroids are steroid hormones that regulate a wide range of physiological processes in plants and influence plant architecture)^12,13^. In barley, the crucial role of BRs in regulating plant growth has been confirmed by molecular characterization of the spontaneous mutant *uzu*. This mutant holds value for hull-less barley breeding in East Asia.^14^ It carries a substitution in the *HvBRI1* (*Brassinosteroid-Insensitive1*) gene encoding the transmembrane BR receptor.^15^ Recently, remarkable advances have been made in elucidating the molecular mechanisms underlying BR signaling.^16–18^ Some components of the BR signaling pathway act as multifunctional proteins involved in other signaling networks that regulate diverse physiological processes, including responses to drought. However, studies on the influence of BRs on plant tolerance to drought are ambiguous. For instance, increased levels of BRs enhanced photosynthesis by elevating ribulose bisphosphate carboxylase/oxygenase (RuBisCO) and nitrate reductase activities in the leaves of *Brassica juncea* exposed to drought.^19^ In contrast, Northey et al.^20^ reported that a reduction in BRs content could improve plant tolerance to drought. Furthermore, Gruszka et al.^21^ demonstrated that barley mutants defective in BR biosynthesis and perception had higher grain weights per plant under drought stress than under control conditions.

Strigolactones (SLs), the youngest plant hormones derived from carotenoids, are involved in the regulation of both aboveground and underground plant architecture, and act as negative regulators of branching.^22,23^ The biosynthesis of SLs is initially catalyzed by carotenoid isomerase D27 and two carotenoid cleavage dioxygenases (CCD), namely CCD7 and CCD8^24^ Recognition and binding of SLs by the receptor D14, which belongs to the *α*/*β* hydrolase protein family, initiates the perception of this hormone.^25^ Interestingly, D14 is the only receptor of SLs identified in barley.^26^ It has been reported that SLs further activate signaling pathways in plants during biotic and abiotic stress conditions.^27^ Studies on different plant species proved that SLs application may improve the resistance to drought^28^ Ha et al.^29^ suggested that the role of SLs as a positive regulator in stress response resulted from its interplay with abscisic acid (ABA). Additionally, Marzec et al.,^30^ demonstrated that barley and Arabidopsis SL-signaling mutants *d14* were sensitive to water deficiency, and that this hyper-sensitivity to stress may be caused by SLs and ABA interaction.

One of the most important traits with a remarkable impact on cereal productivity is the number of productive tillers developed by plants. Shoot branching is regulated by the crown, the first node above the seed. Because both root and shoot meristems are localized in the crown tissues, their viability is essential for plant performance under adverse conditions and for regeneration after stress treatment.^31^ However, the molecular recognition of crown function under stress stimuli remains marginal, including that of barley.

The aim of the present study was to elucidate drought-induced changes in the crowns of barley phytohormone mutants. Our hypothesis was that hormonal disorders caused by mutations in the *HvGA20ox2*, *HvBRI1*, and *HvD14* genes affected the molecular reaction of barley’s crown to drought influencing the plant development under stress. The multivariate reactions of GAs-biosynthesis, BRs-signaling, and SLs-signaling mutants to drought were investigated by large-scale analyses at the transcriptome and proteome levels, as well as by hormone profiling. The effects of stress application on the phenotypes were also evaluated. We assumed that due to the strong connection between strigolactones and branching, the SLs-signaling mutant manifests the most abundant alternations in crowns and phenotype under drought. Finally, components underlaying the core response to drought, independent of the genetic background, were identified.

## 2. Materials and methods

### 2.1. Plant material

Plant material consisted of seven spring barley accessions, including two reference genotypes, Bowman and Sebastian, and genotypes with mutations in genes associated with studied phytohormones (Figure S1, Table 1). The semidwarf near isogenic lines (NILs) represent the previously characterized mutants defective in GA biosynthesis (*sdw1.a* and *sdw1.d* mutants) or BR signaling (*uzu1.a*) (Table 1) and are derived from a collection which was developed by recurrent crossing of original mutants with the Bowman cultivar (Figure S1). Each NIL harbors a restricted and mapped genomic introgression region, specific for a given mutant, in the genetic background of the Bowman cultivar.^32,33^ The set of mutants also included a newly generated double mutant *sdw1.d*/*uzu1.a* for which Bowman was a reference cultivar. Seeds of Bowman and its near isogenic lines were obtained from the Nordic Genetic Resource Center (NordGen, Alnarp, Sweden). Strigolactone mutant *hvdwarf14.d* (*hvd14.d*) was identified using the TILLING platform (*Hor*TILLUS) developed at the University of Silesia in cv. Sebastian background.^26,34^

**Table 1.** Description of plant material.

	Genotype^a^	Characteristics
1	Bowman	Two-rowed U.S. spring-type cultivar used for development of NILs: line BW827 (with *sdw1.a* allele), line BW828 (with *sdw1.d* allele) and line BW885 (carrying *uzu1.a* allele)
2	*sdw1.a* (GA)	NIL line BW827 developed in the genetic background of ‘Bowman’ cultivar, carrying mutation in *HvGA20ox2* gene (allele derived from semi-dwarf mutant induced in cv. Jotun
3	*sdw1.d* (GA)	NIL line BW828 developed in the genetic background of ‘Bowman’ cultivar, carrying mutation in *HvGA20ox2* gene (allele derived from cv. Diamant)
4	*uzu1.a* (BR)	NIL line BW885 line developed in the genetic background of ‘Bowman’ cultivar, characterized by defect in BR signaling pathway caused by mutation in the *HvBRI1* gene (spontaneous mutation derived from cv. Baitori 11)
5	*sdw1.d*/*uzu1.a* (GA/BR)	A double mutant obtained through a cross between the mutants *sdw1.d* and *uzu1.a*, identified on the basis of *HvGA20ox2* and *HvBRI1* sequencing data
6	Sebastian	Two-rowed, spring cultivar, the parental cultivar for *Hor*TILLUS TILLING population and thus of *hvd14.d* mutant
7	*hvdwarf14.d* (SL)	Mutant in *HvD14* gene, impaired in strigolactone signaling

^a^The symbol of the hormone whose pathway is disturbed by the mutation is given in brackets.

### 2.2. Abiotic stress application and phenotyping characteristic

Drought stress experiment was conducted in the growth chambers under fully controlled conditions according to Kuczyńska et al.,^35^ with modifications. Seeds were sown in pots (H-LSR 4.5 L; 21 cm in diameter and 20 cm in height) filled with a mixture of loamy soil and peat (3:1, w/w). The loamy soil provided a balanced mix of sand, silt, and clay, offering good nutrient retention and drainage properties for plant growth. Peat, being organic matter, added to the mixture’s moisture retention capabilities and helped create a suitable environment for seed germination and plant development in the pots. Five plants in each pot were grown under optimal conditions: soil moisture above 70% of the field water capacity (FWC), 22/16°C day/night, air humidity of 60–70%, a photoperiod of 16/8 h of day/night. For drought treatment (D) the soil moisture was established at 20% FWC.^36^ Water scarcity was imposed at the tillering stage (23–26 of BBCH code) and maintained for 10 days. The BBCH scale is widely used to identify the phenological development stages of a plant.^37^ The soil moisture in each pot was controlled daily by weighing and volumetrically using the FOM/mts device.^38^ In parallel, the optimal irrigation experiment (C) was carried out in the same place and time.

Biological samples of barley crowns for molecular and hormonal analyses were collected at the early stage of drought (3^rd^ day of stress, T1) and at the end of stress application – late drought (10^th^ day of stress, T2). Four replicates were used for RNA sequencing, whereas profiling of proteins and hormones was done in triplicates. Each replication consisted of crown samples collected from three plants per one pot.

Mature plants were harvested manually (one time point), and plant structure and yield components were evaluated with the distinction between main and lateral stems. In total, 22 traits (T1-T22) were measured as indicated in Table S1A. Phenotypic measurements were based on three biological replicates, each consisted of five plants grown in one pot and presented with average trait values.

### 2.3. Whole-genome expression analysis

Barley crown was sampled from both D and C variants at two time points (T1 and T2) in four replications, as mentioned above. Next, they were frozen using liquid nitrogen and stored at −80°C until analysis. RNA was extracted from crown samples according to Mikołajczak et al.^39^ Briefly, total RNA was extracted using Qiagen RNeasy Plant Mini Kit (Hilden, Germany), and genomic DNA contamination was removed during (on-column DNase digestion, RNase-Free DNase Set, Qiagen), and after RNA isolation (DNase Max Kit, Qiagen). RNA quantity, quality, and integrity were determined according to Mikołajczak et al.^40^ Construction of cDNA library (TruSeq stranded mRNA) and mRNA sequencing were commissioned to Macrogen Inc. (Seoul, Republic of Korea) that employed an Illumina NovaSeq6000 platform with a 150 bp paired-end configuration; the numbers of obtained read pairs were from 22.1 to 40.4 M per sample. One sample was filtered out due to library quality, and data for two samples were detected as outlying. Data for the remaining 109 samples showed acceptable pattern of co-variation (in total and between biological replicates; Figure S2a), and were used for further analysis.

### 2.4. Proteomic profiling

Barley crown samples (about 100 mg) gathered from D and C condition variants at two time points (T1 and T2) in three replications were frozen with liquid nitrogen, ground to a fine powder, and stored at −80°C. Protein extraction was performed according to the Hurkman-Tanaka^41^ protocol. Pierce^TM^ bicinchoninic acid (BCA) protein assay kit (ThermoFisher Scientific, Waltham, MA, USA) was employed for protein quantification. Furthermore, the peptide solution was pre-treated with 100 mM dithiothreitol (DTT) for 5 min at 95°C and 100 mM iodoacetamide for 20 min at room temperature; afterward, it was subjected to ‘in-solution’ digestion with trypsin solution (Sequencing Grade Modified Trypsin, Promega, Madison, WI, USA) overnight. Proteomic profiling was conducted employing a Dionex UltiMate 3000 RSLC liquid chromatograph coupled with Q Exactive high-resolution mass spectrometer with an Orbitrap mass analyzer equipped with H-ESI ion source (ThermoFisher Scientific) following the protocol described by Mikołajczak et al.^42^ Protein extraction failed in samples of *sdw1.d/uzu1.a* collected at T1 and in four other samples. Data for the remaining 74 samples showed acceptable pattern of co-variation (in total and between biological replicates; Figure S2B), and were used for further analysis (normalized abundances of protein in 74 samples were given in Tables S6).

### 2.5. Targeted quantification of phytohormones

Analyses of plant hormones were done according to the method by Dziurka et al.^43^ in three biological replicates. Lyophilized and finely ground (zirconia oxide beads, MM400, Retsch, Kroll, Germany) plant material was weighted (about 10 mg), spiked with stable-isotope labeled internal-standard mixture, and triple extracted with buffer (methanol/water/formic acid, 15/4/1, v/v/v). Collected supernatants were joined and evaporated under N_2_. The residue was diluted with 3% methanol in 1 M formic acid and cleaned on hybrid SPE columns (BondElut Plexa PCX, Agilent, USA) as described previously.^44^ Samples after clean-up were dissolved in 50 µl of acetonitrile and separated on UHPLC apparatus (Agilent Infinity 1260, Agilent, Germany) with use of an Ascentis Express RP-Amide analytical column (2.7 μm, 2.1 mm × 150 mm; Supelco, Bellefonte, PA, USA) in gradient of 0.01% formic acid in both water (A) and acetonitrile (B). From 0 to 14 min, 3–12.5% B and then from 14 to 24 min, 12.5% to 46% B, then column flush-out with 100% B for 1 min and re-equilibration for 8 min at 3% B. Detection was conducted on a triple quadruple mass spectrometer MS/MS (6410 Triple Quad LC/MS, Agilent, USA) with electrospray ionization (ESI). The following conditions were found to be optimal for the analysis: capillary voltage of 4 kV, gas temperature of 350°C, gas flow of 12 l/min, and nebulizer pressure of 35 psi. The measurements were conducted by multiple reaction monitoring (MRM) in positive polarity. MassHunter software was used to control the LC-MS/MS system and data analysis. MassHunter Optimizer was used to optimize MRM parameters. MRM details are given in supplemental Table S2; further technical details are also provided in.^45–47^ A stable isotope-labeled internal standard for phytohormones analyses consisted of [15N4]DHZ, [2H5]ZR, [2H2]GA8, [2H2]GA3, [2H2]GA1, [2H2]GA4, [2H2]GA6, [2H2]GA5, [2H5]IAA, [2H6]ABA, [2H4]SA, (OlChemim, Olomouc, Czech Republic), [2H5]JA, (CND Isotopes, Quebec, Canada), and [2H5]OPDA (Cayman Chem. Comp., Ann Arbor, MI, USA). Quantitation was based on calibration curves acquired for pure standards (OlChemim), considering recoveries of internal standards.

### 2.6. Data analysis

The Morexv3 *Hordeum vulgare* genome assembly^48^ (Ensembl Plants rel. 55; RRID:SCR_008680, http://plants.ensembl.org/index.html) was used as a reference for mRNA-seq based SNP and gene expression analyses. Gene identifiers (IDs) were given in the text without the “HORVU.MOREX.r3” prefix. Annotation of barley genes with respect to the KEGG (RRID:SCR_012773, http://www.kegg.jp.) and the Plant Reactome (https://www.plantreactome.gramene.org/.) pathways was done using the OmicsBox (RRID:SCR_023676, https://www.biobam.com). After removing adapter-related sequences and quality trimming using AdapterRemoval ver 2.1.7 (RRID:SCR_011834, https://github.com/MikkelSchubert/adapterremoval.,^49^ (parameters: –minquality 20, –minlength 50), mRNA-seq reads were mapped in the reference genome sequence using TopHat ver. 2.1.1 (RRID:SCR_013035, http://ccb.jhu.edu/software/tophat/index.shtml.,^50^ (parameters: maximum no. of mismatches = 2, –no-mixed, –no-discordant); the mapping efficiency for samples was 75.2–91.0%. SNP calling in mRNA-seq data pooled from all biological replications separately for seven genotypes, obtained in optimal conditions at T1, was performed using the samtools/bcftools pipeline (RRID:SCR_005227, https://samtools.github.io.)^51^ (filtering parameters: %QUAL >40, MAF > 0.10, DP > 80). Protein translation effects for SNPs were predicted using the Variant Effect Predictor (RRID:SCR_007931) at Ensembl Plants. Reads aligned to transcripts were counted using the featureCounts function in Bioconductor (RRID:SCR_006442, http://www.bioconductor.org/), in R 3.6.1 (RRID: SCR_001905, http://www.r-project.org/., Rsubread library^52^; and the resulting data were subjected to differential expression analysis in DESeq2 ver. 1.22.2 (RRID: SCR_015687, https://bioconductor.org/packages/release/bioc/html/DESeq2.html).^53^ Differentially expressed genes (DEGs) between stressed and control samples were found among the genes characterized by a mean expression of at least 5 units (estimated in DESeq2), with the thresholds |log_2_(FC| >2 and FDR < 0.01. Gene Ontology terms enrichment analysis was performed using the package GOfuncR ver. 1.18.0 in R 4.2.2 (Grote S. 2022).^54^

Analyses of proteomic profiling data were performed using MaxQuant 2 (1.5.3.1) (RRID:SCR_014485, https://www.maxquant.org/maxquant/) and Perseus (1.4.1.3) (RRID:SCR_015753, https://www.maxquant.org/perseus/) software together with Proteome Discoverer 2.2 (ThermoFisher Scientific) (RRID:SCR_014477, https://www.thermofisher.com/pl/en/home/industrial/mass-spectrometry/liquid-chromatography-mass-spectrometry-lc-ms/lc-ms-software/multi-omics-data-analysis/proteome-discoverer-software.html). Protein libraries were explored handling the SequestHT tool for proteins prepared for *Hordeum vulgare* in the UniProt database (RRID:SCR_002380, https://www.uniprot.org). Differential abundance of proteins was declared at FDR < 0.05.

Phenotypic data were subjected to analysis of variance in the model containing fixed effects of genotype, watering treatment and their interaction, with estimation of contrasts (treatment effects) and testing their significance by the *t* test (at Bonferroni-corrected *p* < .05). Phytohormone data were analyzed in the same way after log_2_ transformation and with the inclusion of factor “time of observation”, with corresponding interactions, in the analysis of variance. These analyses, as well as hierarchical clustering of genotypes using appropriate similarity matrices and algorithms (see Results), were conducted in Genstat 19 (RRID:SCR_014595, http://www.vsni.co.uk/products/genstat/).^55^

A weighted gene co-reaction network analysis was performed using the WGCNA library (RRID:SCR_003302, http://www.genetics.ucla.edu/labs/horvath/CoexpressionNetwork/.) in R^56,57^ with parameters: beta = 6, complete link clustering method, cutHeight = 0.98, minsize = 10.

## 3. Results

Multiomics examination of seven barley genotypes was performed. To illustrate their behavior under transitory drought (T1, T2) genotypes were clustered on the base of each single data type (Figure S3).

### 3.1. Single nucleotide polymorphism (SNP) of genotypes

Hierarchical clustering based on a kinship matrix computed from 46,170 polymorphic markers (homozygous) between genotypes, revealed by SNP calling from RNA-seq data, confirmed the close similarity between the mutants and their reference genotypes (Figure S3a, Table S3). Notably, among all the NILs, *sdw1.a* and *sdw1.d* mutants (GA disorders) showed the greatest and smallest genetic similarity to Bowman, respectively. A total of 45,456 SNPs that affected protein translation were found using the VEP tool (Table 2). Most SNPs of HIGH effect (89) were found in stop codons (‘stop gained’ or ‘stop lost’ variants), while SNPs of MODIFIER effect were numerously present in introns and 3’ UTR, and to a lesser extent in 5’ UTR (Table S3).

**Table 2.** Number of SNPs between genotypes with effect on protein translation assigned by VEP tool.

Number of SNPs	Effects predicted by VEP (Ensembl Plants) for 45,456 SNP			
	HIGH	LOW	MODERATE	MODIFIER
46170	89	17756	11601	16010

### 3.2. Differentially expressed genes (DEGs)

**Figure 1.**
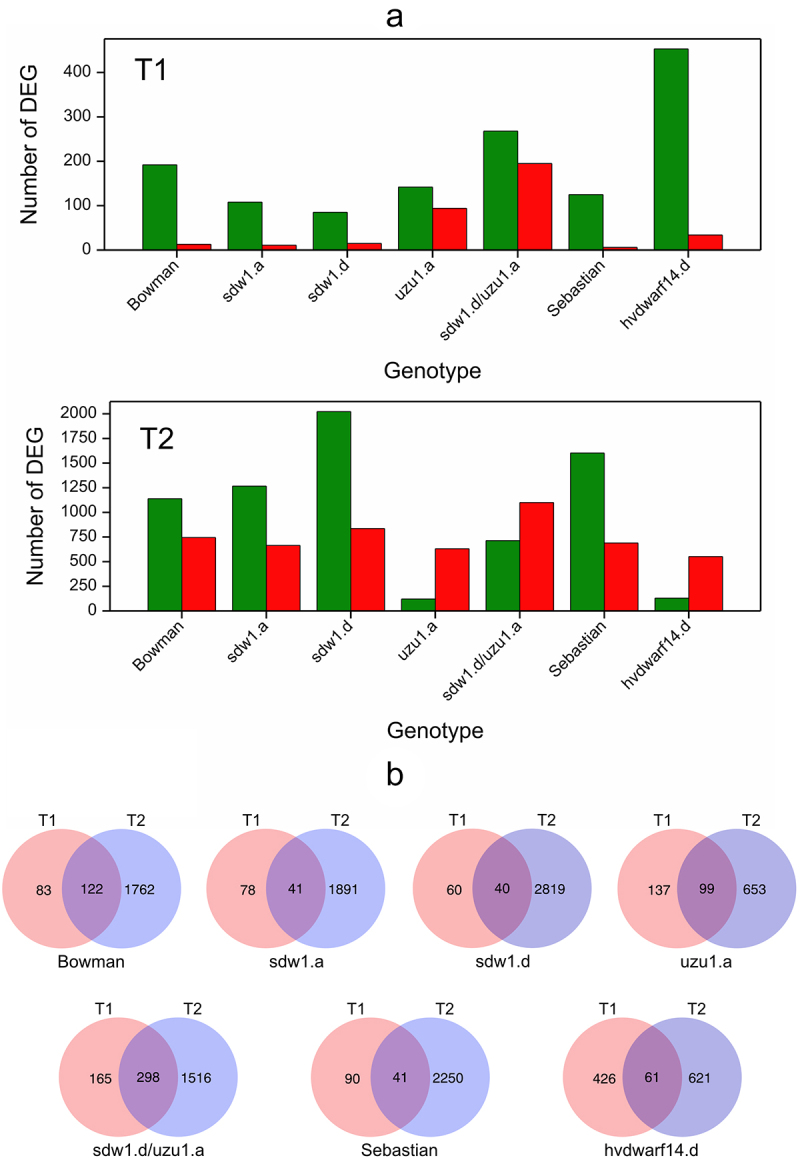
(a) Number of down- (green) and upregulated (red) genes for seven genotypes in drought vs. control comparison at two time points T1 and T2, (b) Venn diagrams visualizing the number of differentially expressed genes in crown tissue for seven genotypes specific and shared between time points T1 and T2.

Differential gene expression analysis showed that more changes in transcript abundance were observed at the end of the drought treatment than at the beginning (Figure 1, Table S4). Genes with reduced expression (in relation to the control) predominated at T1 in most genotypes. Notably, at T2 the majority of DEGs were upregulated in *uzu1.a* (BR), *sdw1.d/uzu1.a* (GA/BR), and *hvdwarf14.d* (SL), whereas in other cases, the “downregulation” was predominant. Clustering of genotypes based on estimates of contrasts between drought and control conditions (log2FC values) for DEGs that were significant in at least one contrast showed a close similarity in the transcriptomic response between *uzu1.a* and *hvdwarf14.d* (Figure S3b) at T2. Concurrently, the profiles of the transcriptomic responses of these two genotypes were different from the profiles observed for their respective reference genotypes at T2. At T1, the Bowman and Sebastian transcriptomes were similar to those of *uzu1.a* and *hvdwarf14.d*, respectively. Interestingly, the pattern of gene expression changes in *sdw1.d/uzu1.a* mutant was distinct from that of all other genotypes.

A set of DEGs affected by both early and late drought was identified in all genotypes. Among them, some DEGs (from 1 for *sdw1.d/uzu1.a* to 38 for *hvdwarf14.d*) changed the direction of the reaction between early and late drought in all genotypes, except Bowman. Generally, these genes were initially downregulated under drought conditions compared to the control, and they showed increased expression during prolonged stress (Table S4). The largest number of these DEGs was identified for the *hvdwarf14.d* mutant. Out of these, five genes of altered regulation over time of stress were annotated to phosphorylation and seven genes were involved in hormones signaling pathway or biosynthesis (according to Plant Reactome database), namely ABA, SA, and JA. Next, we extracted DEGs identified in at least 10 “contrasts” across genotypes and time points as candidates of universal genes for plant functioning under drought. Most were affected by both early and late drought in the studied genotypes, excluding Sebastian and *hvdwarf14.d* (SL), where late-responsive genes were predominant (Table S4). Among genotype-universally differentially expressed genes, several DEGs corresponded to carbohydrate metabolism (“downregulation” across genotypes excluding 3HG0230420), LEA proteins (“upregulation” with some genotypes except for *sdw1.a* (GA) during early drought) and lipids (fatty acid biosynthesis and lipolytic enzyme GDSL lipase/esterase-downregulation, and lipids transport-upregulation).

Gene Ontology (GO) terms enrichment analysis was used for the functional interpretation of the detected DEGs (Table S5). In general, this analysis showed that the DEGs in *hvdwarf14.d* (SL) and Sebastian differed functionally in response to drought, as very few GO terms were shared between the two genotypes at each time point. For instance, several GO terms related to the cell wall enriched in the Sebastian at T2 were not observed in *hvdwarf14.d*. Similarly, these terms were enriched in Bowman and *sdw1.a* and *sdw1.d* mutants (GA disorders) but not in the *uzu1.a* (BR) at T2. Conversely, GO terms associated with oxidoreductase activity were overrepresented in *sdw1.a* and *sdw1.d* mutants at T2, in *uzu1.a* at T1, and in Bowman at both time points. These terms were inconsistently enriched in *hvdwarf14.d* and Sebastian, namely at T1 and T2, respectively. Overall, most of the overrepresented GO terms in *hvdwarf14.d* during the early drought were not enriched until the late drought in Sebastian. Generally, GO terms associated with the response to various stimuli/compounds (such as oxidative stress and toxic substances) were enriched under late drought, being most abundant in *uzu1.a* (GA) and *hvdwarf14.d* (SL) and least abundant in *sdw1.d/uzu1.a* (GA/BR). Notably, the term “response to abscisic acid” was overrepresented specifically in *uzu1.a* and *hvdwarf14.d* at T2, whereas four terms related to phosphorylation were enriched exclusively in Sebastian, mostly at T1. Most of the terms enriched in the *sdw1.d/uzu1.a* mutant during early drought were coincident with the terms of *uzu1.a*, and only two of these terms were found in the *sdw1.d* mutant.

#### 3.2.1. Expression analysis of genes related to phytohormones and drought

To learn more about the behavior of genes in crowns exposed to drought and its association with main hormone classes affecting branching, namely GAs, BRs, and SLs, we extracted DEGs annotated to these hormones by searching for hormone names in gene description, gene GO annotation, and gene annotation by pathways (Table S4). In total, 51, 28, and 21 DEGs were associated with signaling/biosynthesis of GAs, BRs, and SLs, respectively (Table S4). The number of DEGs related to the hormones of interest was markedly lower in *uzu1.a* (BR) and *hvdwarf14.d* (SL) than in other genotypes. Generally, late drought stress induced more changes in the expression of these genes than early drought stress (Table 3). The expression levels of *HvGA20ox2*, *HvBRI1*, and *HvD14*, mutated or not, were unaffected by drought (Table S4).

**Table 3.** Number of DEGs related to GA, BR, SL, and drought response identified in seven genotypes under early (T1) and late (T2) drought.

Genotype	Time point	Number of DEGs annotated to:			
		GA	BR	SL	Drought
Bowman	T1	1	3	0	2
	T2	27	11	6	24
*sdw1.a*	T1	0	0	0	2
	T2	20	12	10	23
*sdw1.d*	T1	2	0	0	1
	T2	25	21	11	27
*uzu1.a*	T1	1	2	0	7
	T2	5	2	2	16
*sdw1.d/uzu1.a*	T1	4	1	0	10
	T2	21	10	6	26
Sebastian	T1	2	0	0	0
	T2	24	17	10	28
*hvdwarf14.d*	T1	4	1	1	4
	T2	2	2	2	13

##### 3.2.1.1. Gibberellin-related genes

**Figure 2.**
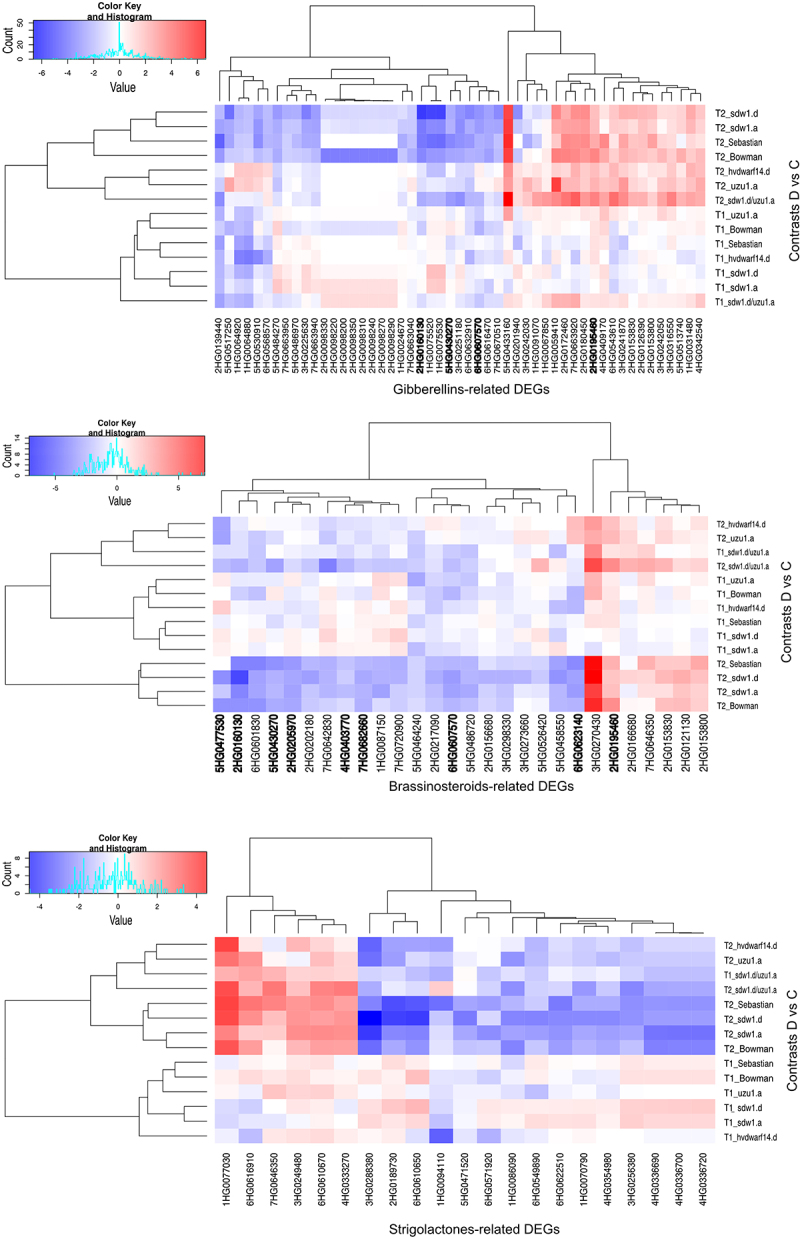
Heatmap of log2FC values with grouping of differentially expressed genes (DEGs) related to gibberellins (GAs), brassinosteroids (BRs), and strigolactones (SLs) in drought and control comparison (contrasts D vs C) for seven genotypes in two time points (T1, T2); DEGs annotated to transcription factor bHLH family are in bold.

Approximately half of the GA-related DEGs identified in Bowman were present in *sdw1.a* (GA) and *sdw1.d* (GA) at T2 and were largely downregulated (Table S4). However, 10 genes related to GA signaling, which showed decreased expression in Bowman at T2, were unaffected in NILs. In turn, five DEGs showed reduced expression in response to late drought in both *sdw1* NILs but not in Bowman. One such gene, 5HG0517250, which is involved in the GA signaling pathway, showed enhanced expression in *uzu1.a* (BR) during late drought. Six DEGs identified in *sdw1.d* were not differentially expressed under drought in *sdw1.a* or in Bowman; three of which, with enhanced expression in response to drought at T2, were also detected in *sdw1.d/uzu1.a* (GA/BR), including 1HG0031480, annotated to gibberellin 2-oxidase. In *sdw1.d/uzu1.a*, approximately 4-fold more GA-related DEGs were upregulated than downregulated, whereas in *uzu1.a* (BR), no GA-related genes showed decreased expression under drought conditions. The gene 1HG0091070, which is involved in GA biosynthesis, was specifically upregulated in *sdw1.d/uzu1.a* under late stress. Overall, the expression profile of GA-related DEGs in *sdw1.d/uzu1.a* was the same as that in *sdw1.d* for half of these genes and was substantially different from that of the *uzu1.a* mutant. GA-related DEGs detected in *hvdwarf14.d* (SL) during early and late drought were downregulated and upregulated, respectively. In total, four of these DEGs were similar to those in Sebastian, whereas 6HG0568570, the rice ortholog gene encoding the WRKY71 TF, was exclusively downregulated in *hvdwarf14.d*. In contrast, 21 DEGs were exclusively affected in Sebastian at T2 compared to in *hvdwarf14.d* (Figure 2, Table S4).

##### 3.2.1.2. Brassinosteroid-related genes

Most of the drought-responsive genes associated with BR signaling were downregulated in Bowman (primarily at T2) and were also identified in *sdw1* NILs (GA disorders) but were not found in the *uzu1.a* (BR) mutant. Only two of these genes were common between Sebastian and *hvdwarf14.d* (SL). The behavior of the BR-related DEGs in *sdw1.d/uzu1.a* (GA/BR) was more similar to that observed in *sdw1.d* than that in *uzu1.a*. The gene 3HG0270430 (assigned to the DnaJ domain) was universally upregulated in response to drought across genotypes, mainly in T2. Most BR-related DEGs were annotated to the domains of various transcription factors, primarily (nine genes) to the basic helix-loop-helix (bHLH) (Table S4). All genes associated with bHLH showed decreased expression, especially under late drought in Bowman, *sdw1* NILs, and Sebastian, except for 2HG0195460, whose expression was enhanced at T2 in Bowman, Sebastian, and *sdw1.d/uzu1.a*. One such drought-induced gene, 4HG0403770, was specific to *sdw1.d/uzu1.a*. Interestingly, four bHLH-related DEGs were affected by drought, including rice ortholog genes encoding bHLH79 and bHLH101 (according to Ensembl Plants), corresponding to the signaling of both brassinosteroids and gibberellins, three of which were found, among others, in *sdw1.d/uzu1.a* and *sdw1* NILs, and none in the *uzu1.a* mutant. Two additional late drought-responsive DEGs were involved in GA and BR signaling, namely, 2HG0153830 in the *sdw1.d* and *sdw1.d/uzu1.a* mutants, and 2HG0153800 in Sebastian. Both genes were annotated to aldo – keto reductase and overexpressed under drought conditions in T2 compared to the control. Three DEGs were annotated to the WRKY domain, one of which, 5HG0464240, a rice ortholog gene encoding WRKY50, was specifically downregulated in the *uzu1.a* mutant under early stress and was annotated to JA signaling. Two BR-related DEGs corresponded to GRAS TFs, and both showed reduced expression in response to late drought in *sdw1.d* (GA) and Sebastian, whereas 7HG0642830 was additionally downregulated in *sdw1.a* (GA) and *sdw1.d/uzu1.a* (GA/BR) at T2. The gene 2HG0202180, which encodes the transcription factor IBH1-like, was downregulated exclusively in *sdw1.d* and Sebastian under prolonged drought. Curiously, we identified a DEG putatively encoding BKI1 (an inhibitor of receptor kinases), whose expression was reduced in *sdw1* NILs and Sebastian (Figure 2, Table S4).

##### 3.2.1.3. Strigolactone-related genes

All DEGs related to SLs showed changed expression (mainly “downregulation”) only in response to late drought across genotypes (with one exception for *hvdwarf14.d*). The largest number of SL-related DEGs was found for *sdw1* NILs (GA disorders) and Sebastian, whereas it was the lowest for the *uzu1.a* (BR) and *hvdwarf.14d* (SL) mutants (Table 3). Primarily, these DEGs corresponded to SLs signaling and were the most numerously annotated (eight DEGs) to the ubiquitin-conjugating enzyme E2; notably, three of them were exclusively underexpressed in the *sdw1.d* mutant (Table S4). Among the DEGs involved in SLs signaling, 1HG0077030, annotated to the ClpA/B family, was overexpressed under late drought conditions in all genotypes. In contrast, among the DEGs related to SLs biosynthesis, 3HG0288380, which is associated with carotenoid oxygenase, showed reduced expression across genotypes, excluding *sdw1.d/uzu1.a* (GA/BR). Interestingly, three DEGs annotated to the TCP transcription factor showed decreased expression under drought in T2 only in Bowman and *sdw1.a*. One drought-responsive DEG (7HG0646350) was upregulated in *sdw1.d/uzu1.a* and Sebastian, which corresponded to the signaling of SLs, BRs, and JA (Figure 2, Table S4).

##### 3.2.1.4. Drought-related genes

**Figure 3.**
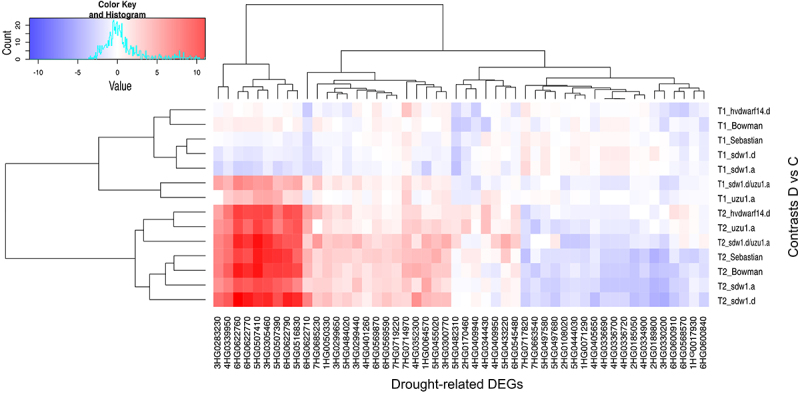
Heatmap of log2FC values with grouping of differentially expressed genes (DEGs) related to drought in drought and control comparison (contrasts D vs C) for seven genotypes in two time points (T1, T2).

To better understand the behavior of genes related to the drought response in hormone-perturbed genotypes and their reference genotypes, 50 DEGs annotated with drought/water deprivation were identified (Table S4). They were most affected by the late drought, with *hvdwarf14.d* (SL) and *uzu1.a* (BR) mutants possessing the lowest number of DEGs. Meanwhile, the early drought induced the most changes in gene expression, namely “upregulation,” in the *sdw1.d/uzu1.a* (GA/BR) and *uzu1.a* lines (Table 3). Primarily, they corresponded to dehydrin (nine genes) and were overexpressed during late drought in all genotypes (Table S4). Interestingly, one of them, 6HG0622710, showed a contrasting regulation in *hvdwarf14.d* between early and late drought, namely “downregulation” and “upregulation,” respectively. A similar behavior of 6HG0622760 was observed in *sdw1.a* (GA). Among the genes induced by stress in Sebastian and unaffected in *hvdwarf14.d* (SL), eight DEGs were related to kinases. The reaction of drought-related genes in Bowman was similar to that observed in its three NILs, especially *sdw1.a*; however, the three DEGs annotated as TCP were unaffected by *sdw1.d* (GA) and *uzu1.a* (BR), as indicated above. Of note, 13 DEGs were annotated to the AP2/ERF domain, and the regulation of their expression under drought conditions was inconsistent. Five of them were genotype-specific, including *sdw1.a* (one downregulated DEG), *sdw1.d* (two downregulated DEGs), *uzu1.a* (one upregulated DEG), and *sdw1.d/uzu1.a* (one upregulated DEG). In *sdw1.d/uzu1.a*, gene 3HG0252020 changed its regulation from upregulation during early drought to downregulation during late stress. This DEG is involved in the HSFA7/HSFA6B-regulatory network induced by drought and ABA. Among the DEGs annotated to the drought response, one gene was related to GA signaling (corresponding to WRKY TF) and three to SL signaling (corresponding to TCP TFs), as mentioned above (Figure 3, Table S4).

### 3.3. Differentially abundant proteins (DAPs) and comparison of protein and gene expression

**Figure 4.**
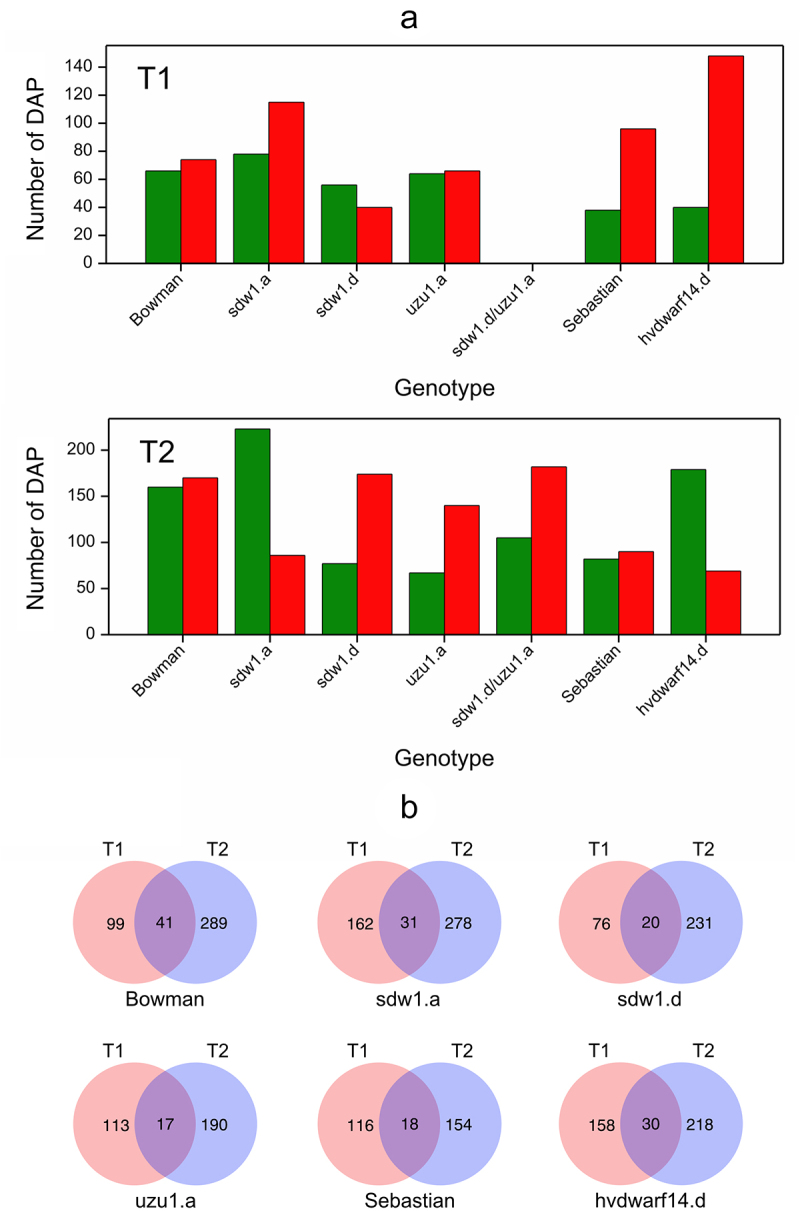
(a) Number of down- (green) and upregulated (red) proteins for seven genotypes in drought vs. control comparison at two time points T1 and T2, (b) Venn diagrams visualizing the number of differentially abundant proteins in crown tissue for seven genotypes specific and shared between time points T1 and T2.

Overall, 1,392 differentially abundant proteins were identified in at least one of the 14 analyzed comparisons between the drought and control conditions (Table S6). Late drought stress induced more changes in protein accumulation than early drought stress (Figure 4). Curiously, in *hvdwarf14.d* (SL), almost 4-fold more DAPs had increased accumulation than reduced under early stress, whereas under late drought, a higher number of downregulated proteins were found (2.5-fold). In *sdw1.a* (GA), upregulated DAPs were initially more numerous, but with prolonged drought, downregulated DAPs predominated; in the *sdw1.d* (GA) mutant, the situation was opposite – there were more “downregulations” and “upregulations” in T1 and T2, respectively. A relatively small fraction of DAPs (approximately 10% in Bowman) was common between the early and late drought periods in each genotype. Among the DAPs that changed their direction of regulation over time under stress (from 8 for Sebastian to 16 for *sdw1.a*), three were common between the two genotypes (Table S7). F2DQK4 (O-methyltransferase COMT-type) was upregulated and downregulated in *hvdwarf14.d* and *sdw1.a* mutants at T1 and T2, respectively. A similar behavior was observed for F2CSS7 (glycoside hydrolase) in *hvdwarf14.d* (SL) and *uzu1.a* (BR) mutants, whereas the direction of regulation of A0A287PYF7 (unknown) was opposite between these two genotypes at each time point.

**Figure 5.**
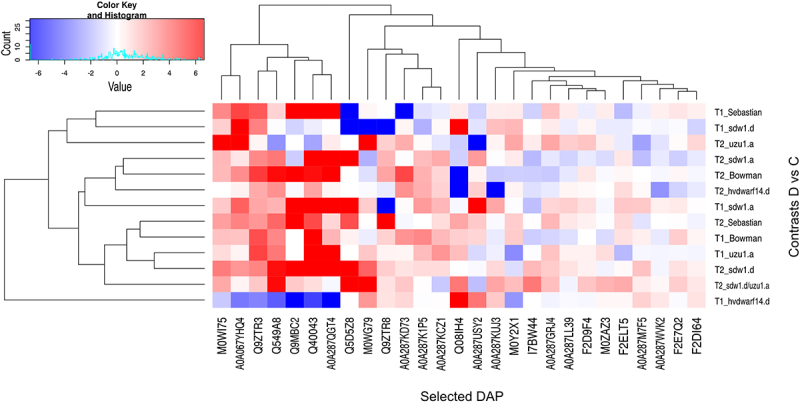
Heatmap of log2FC values with grouping of selected differentially abundant proteins (DAPs) in drought and control comparison (contrasts D vs C) for seven genotypes in two time points (T1, T2).

Clustering of genotypes based on log2FC values for DAPs demonstrated the close similarity of proteomes between *sdw1.a* and *sdw1.d* mutants under early stress; however, their proteomic response to late drought was distinct (Figure S3c). The profiles of proteomic changes in *hvdwarf14.d* (SL) and its reference genotype Sebastian were substantially dissimilar over the duration of stress, and the proteomic reaction of *hvdwarf14.d* to late drought was most similar to the response of *sdw1.a*, whereas those of Sebastian were close to those of *sdw1.d*. The proteomic landscape of *sdw1.d/uzu1.a* (GA/BR) at T2 was slightly more similar to that of *sdw1.d* (GA) than to that of the *uzu1.a* (BR) mutant. Next, we analyzed whether some proteins known to participate in the stress response were affected by drought in the mutants, but not in their respective reference genotypes, and *vice versa*. Among the DAPs identified in *hvdwarf14d* and not observed in its reference genotype, Sebastian, there were, for instance, (i) the early drought-responsive upregulated proteins: rubrerythrin domain-containing protein (A0A287KUJ3) or sHSP domain-containing protein (A0A287USY2); (ii) the late drought-responsive proteins: pyrroline-5-carboxylate reductase (F2E7Q2) and tryptophan synthase (A0A287WVK2) – downregulated, and two sHSP domain-containing proteins (A0A287K1P5, A0A287KD73) – upregulated (Figure 5, Table S6). Meanwhile, two WHy domain-containing proteins (A0A287GRJ4, M0WI75) were overaccumulated in Sebastian (unaffected in *hvdwarf14d*) regardless of the time of stress exposure. Several proteins involved in sugar metabolism and sHSP domain-containing protein (A0A287KCZ1) were upregulated, whereas the malic enzyme (F2ELT5) was downregulated in *uzu1.a* but was not induced by drought in Bowman. In contrast, we found that the HVA22-like protein (A0A287QGT4) and five dehydrins (Dhn 1, 4, 7, 9, and 11) were overaccumulated in Bowman under early and late drought, respectively, and were not induced in *uzu1.a*. Similarly, dehydrin 9 (Q9ZTR3) was upregulated under drought in Bowman but was unaffected in *sdw1.a*. and *sdw1.d*. In contrast, dehydrin 7 (Q5D5Z8) showed increased accumulation in *sdw1.a* exclusively. Interestingly, 25 DAPs at T2 were shared between both *sdw1* NILs, but mostly they had opposite regulatory statuses, including 1-aminocyclopropane-1-carboxylate (ACC) (I7BW44) and calreticulin (A0A287M7F5), which were both down- and upregulated in *sdw1.a* and *sdw1.d*, respectively. Among drought-induced proteins in *sdw1.d/uzu1.a*, which were unaffected in *sdw1.d* and *uzu1.a*, we found, for instance, aquaporin (Q08IH4), methionine S- methyltransferase (Q9MBC2), and chalcone- flavonone isomerase (M0ZAZ3) upregulated (Figure 5, Table S6).

Integration of proteomic and transcriptomic data revealed that 1,467 gene identifiers were assigned to proteins through the presence of the UniProt TrEMBL protein identifier within the annotation of genes (Table S8A). Interestingly, in 34 cases of them, the direction of gene and protein regulation was consistent in at least one and the same “contrast,” primarily under the late drought (Table S8B). The largest number of coincident regulation cases was found for pairs 1HG0049040-F2D3S4 and 5HG0441580-A0A287QL18, both of which are involved in the HSFA7/HSFA6B-regulatory network induced by drought and ABA (according to the Plant Reactome database) and for 7HG0707080-M0VJA1 (seed maturation protein 1). Generally, they were upregulated in Bowman, *sdw1* NILs, and Sebastian during the late drought. However, the second pair was also upregulated in *uzu1.a* (BR) during the early drought; notably, all proteins within these pairs were downregulated in *hvdwarf14.d* (SL) at T1. The pair 3HG0248140-M0WI75 (LEA 2) was upregulated in *sdw1.d* (GA), *sdw1.d/uzu1.a* (GA/BR), and Sebastian at T2. Two dehydrins were overexpressed together with the corresponding genes at T2: one in Bowman and *sdw1.a* (GA) and the second in *sdw1.d* and Sebastian. The GO terms enrichment analysis (corrected *p* < .05) of DEGs paired with DAPs having consistent regulation confirmed the overrepresentation of “response to water” and “response to acid chemical” GO terms.

### 3.4. Phytohormones profiling

**Figure 6.**
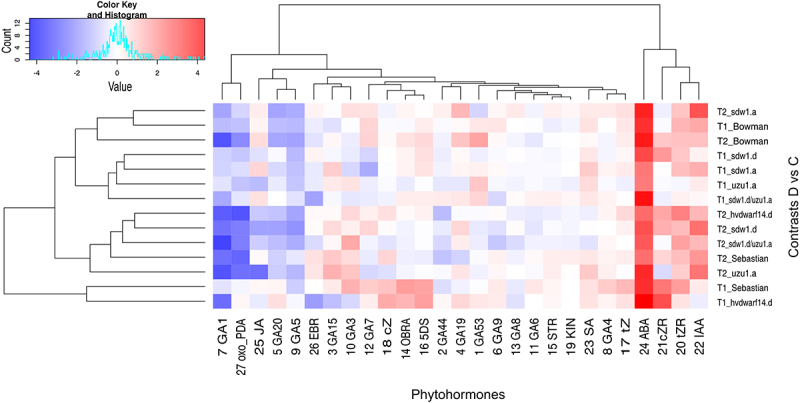
Heatmap of log2FC values with grouping of phytohormones in drought and control comparison (contrasts D vs C) for seven genotypes in two time points (T1, T2).

Measurements of the qualitative and quantitative composition of the plant hormones in the crown included the analysis of 27 selected phytohormones, mainly GAs, BRs, and SLs, as well as auxins, cytokinins (CK), and others, which were examined at T1 and T2 (Table S9). Bowman, *uzu1.a* (BR) and *sdw1* NILs (GA disorders) showed similar overall hormonal responses to early drought (Figure S3d); however, there were significant differences in the reactions of the selected hormones (Figure S4). For instance, the levels of GA_1_ and GA_20_ were reduced in Bowman at T1, whereas they remained unchanged in the aforementioned mutants. In response to late drought, the hormonal profiles of these genotypes became more distant. Again, the reaction of *uzu1.a* was more distinct from the response of Bowman than that of *sdw1* NILs, independent of the time point. An increase in GA_53_ accumulation was observed only in Bowman under drought conditions (T2). The level of SA increased considerably only in *uzu1.a* under late drought conditions. Drought-induced changes in *sdw1.d/uzu1.a* (GA/BR) mutant were substantially different from those observed in Bowman and other NILs at T1, whereas at T2, the hormonal profiles of the double mutant and *sdw1.d* were more similar to those of Bowman. The *sdw1.d/uzu1.a* mutant was distinguished by the behavior of gibberellins in response to late drought, namely an increased abundance of GA_3_ and decreased levels of GA_8_ and GA_44_, whereas their levels did not change in Bowman and other NILs (Figures 6 and S4). The epibrassinolide (EBR) content was reduced in response to early stress only in the double mutant and *hvdwarf14.d* (SL). The *sdw1.d* had increased accumulation of cis-zeatin riboside (cZR) at both time points, whereas *sdw1.a* showed increased level of trans-zeatin (tZ) at T2. In turn, a reduced cis-zeatin (cZ) content was found in *uzu1.a* (BR) at T2. All the above-mentioned cytokinins showed increased accumulation in *hvdwarf14.d* (at both time points) and Sebastian (mostly at T1).

A remarkable effect of water treatment on the accumulation of strigolactones, namely orobanchol (OBRA) and 5-deoxystrigol (5DS), was observed only in Sebastian and *hvdwarf14.d* at T1 (Table S9, Figure S4). However, the overall hormone remodeling in Sebastian and *hvdwarf14.d* was substantially different under early and late stress conditions (Figure S3d) and mainly corresponded to the abundance of gibberellins. Early stress caused an increase in GA_19_ content in *hvdwarf14.d*, whereas it was reduced in Sebastian in response to late drought. Additionally, GA_3_ and GA_4_ overaccumulated exclusively in Sebastian in the drought vs. control (T1), whereas the abundance of GA_5_ and GA_20_ was reduced in the mutant at T2 and did not change in Sebastian. Increased accumulation of ABA in response to drought was observed in all genotypes. Interestingly, over the drought time, that is, from the 3^rd^ (T1) to the 10^th^ (T2) day of stress, the ABA content increased in Bowman, NILs, and the double mutant, whereas it decreased in Sebastian and *hvdwarf14.d* (Figures 6 and S4). Across all genotypes, no effect of drought was observed on the accumulation of GA_6_, GA_9_, GA_15_, strigol (STR), and kinetin (KIN) at either time point. Marked mean genotype, water treatment, and time-point effects were identified for gibberellins including GA_1_, GA_3_, GA_8_, GA_19_, GA_20_, GA_44_, and GA_53._ For OBRA, a significant effect was not detected only in the case of the time points. Conversely, for EBR, a significant effect of time point (day of stress) or genotype × time point × treatment interaction was not observed (Table S9A). Overall, the genotype-specific drought effects mentioned above were confirmed by the significance of the G × W interaction for the 13 phytohormones, and the time-specific effects were confirmed by the significance of the W × T interaction for the same number of traits.

### 3.5. Co-reaction network of genes, proteins, and hormones affected by drought

**Figure 7.**
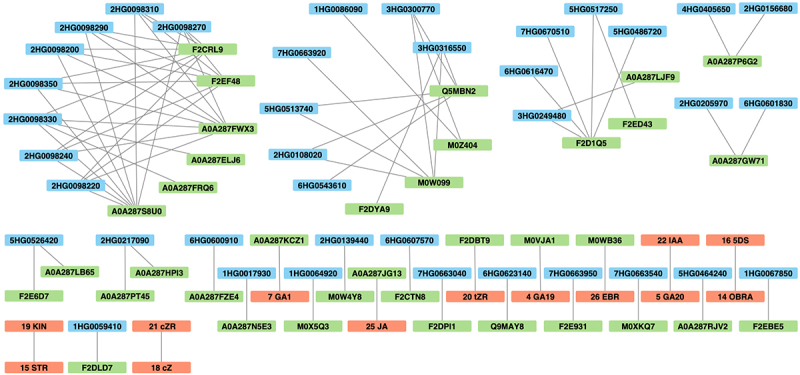
The network of differentially expressed genes related to GAs, BRs, SLs (blue), differentially abundant proteins (green) and hormones (red) with substantially correlated expression profiles (adjacency coefficient > 0.3).

To elucidate the relationships between the reactions of genes (related to GA, BR, SL, and drought), proteins, and hormones affected by drought, a co-reaction network was constructed using data consisting of (drought – control) contrast values for seven genotypes at two time points (Figure 7, Table S10). Network edges between the same type of data were filtered out, excluding hormone–hormone relationships. Numerous connections have been observed between DEGs and DAPs, particularly for GA-related genes, indicating correlated responses. Proteins with the greatest number of significant connections with genes were mostly uncharacterized. However, Pfam annotation revealed that one such protein, F2EF48 (Pfam ID: PF00011), could represent the heat shock factor HSP20. Additionally, the activity of the sHSP domain-containing protein (A0A287KCZ1) was correlated with that of GA_1_ under drought conditions. Three GA-related DEGs had reactions correlated with thaumatin (Q5MBN2) activity under drought conditions, and two of them were additionally associated with M0W099 (unknown protein), which, according to the Pfam entry (PF00314), also represents thaumatin. BR-related DEGs were associated with several enzymes, including glutamine amidotransferase type-2 domain-containing protein (A0A287LB65). Among the proteins connected with DEGs annotated to the drought response, two represented thaumatin, as described above, and one of these genes encoded the basic-leucine zipper (bZIP) transcription factor. One of the SL-related genes correlated with peroxidase (F2D1Q5) activity under drought conditions. Interestingly, we also identified an interaction between glucose-6-phosphate 1-epimerase (A0A287JG13) and JA. Remarkable interactions between different types of hormones affected by drought were found for GA_20_ and indole-3-acetic acid (IAA) and for STR and KIN.

### 3.6. Effect of drought on phenotypes

**Figure 8.**
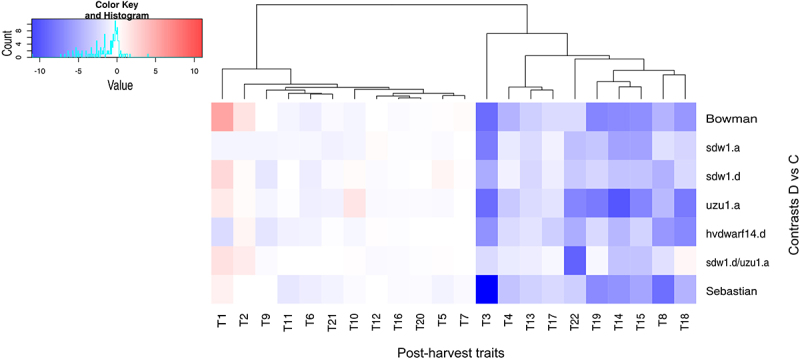
Grouping of post-harvest traits (T1–22; see table S1A) in drought and control comparison (contrasts D vs C) for seven genotypes.

The ANOVA revealed considerable effects of genotype on all observed phenotypic traits (Table S1A). Water treatment also had a significant effect on all traits except for the length and number of internodes of the main and lateral stems (*p* < .01). For 14 of the 22 traits, the genotype × water treatment interaction was considerable, which was equivalent to the existence of genotype-specific phenotypic drought effects. Overall, for all genotypes, drought caused a reduction in the length of the main spike and the number of spikelets per main spike, but no effect was detected on the bottom internode length of the main stem or the number of internodes of the main and lateral stems (Table S1B and S1C). A much higher increase in tiller number was detected under drought conditions in Bowman than in the other genotypes (Figures 8 and S5). Only *hvdwarf14.d* (SL) showed a marked reduction in the number of tillers per plant under drought conditions, similar to the peduncle length of the lateral stem. Interestingly, *sdw1.d/uzu1.a* (GA/BR) was the only genotype in which water deficit did not cause significant changes in the length of the main stem. The length of lateral stem and spike and the number of spikelets and grains per lateral spike were also not affected by stress treatment in *sdw1.d/uzu1.a*, as well as in *sdw1.a* (GA) and *sdw1.d* (GA). Across all genotypes, only Sebastian and its mutant *hvdwarf14.d* were characterized by a considerably reduced internal internode length of the lateral stem under drought. Both reference genotypes and *uzu1.a* showed the greatest stress-induced reduction of grain number and weight of main and lateral spikes. The grain weight per plant was reduced by drought in *sdw1.a* and *sdw1.d* mutants but not in their reference genotype, Bowman, whereas the trait value was decreased in Sebastian but not in its mutant *hvdwarf14.d* (Figures 8 and S5). Moreover, the grain weight per main spike did not change significantly under unfavorable environmental conditions for *hvdwarf14.d*. Meanwhile, the thousand grain weight was reduced by drought in the three above-mentioned mutants but was unaffected by either reference genotype. A dendrogram based on the values of (drought – control) contrasts for all phenotypic traits revealed a higher similarity of phenotypic performance between Bowman and Sebastian (Figure S3e). Moreover, Sebastian was more similar to the *uzu1.a* (BR) mutant, whereas *hvdwarf14.d* (SL) was more similar to the *sdw1* NILs (GA), indicating that mutants *sdw1.a* (BR), *sdw1.d*, and *hvdwarf14.d* represented the most different phenotypes compared with the respective reference genotypes. The behavior of phenotypic traits under drought in *sdw1.d/uzu1.a* (GA/BR) was rather different from that observed for *sdw1.d* or *uzu1.a* mutants.

## 4. Discussion

Currently, data on the molecular characterization of barley crowns are scarce, especially from large-scale studies. Herein, we present our second study on this part of the plant, expanding our knowledge about the multifaceted behavior of barley crowns under exposure to transitory drought. One of the unique aspects of the present study was the experimental design, which allowed us to compare the drought-induced reactions of barley varieties and mutants with genes that determine the production/signaling of different phytohormones, namely gibberellins, brassinosteroids, and strigolactones. Statistical data analysis was directed at estimating and testing the effects of drought (contrasts) for all observed traits, separately for all studied genotypes, and for transcriptomic, proteomic, and phytohormone data, separately for two time points. This allowed us to identify situations in which the effects were genotype- or time-specific owing to the interactions of the factors used in the experiment. General hypotheses about these interactions were tested for phytohormone and phenotypic data, but not for transcriptomic and proteomic data, as this would lead to the reporting of too many results.

Our research showed that, despite the large genetic distance between Bowman and Sebastian, they were the most similar to each other at the phenotypic level, as were *hvdwarf14.d* (SL) to *sdw1.d* (GA). These discrepancies between phenotypes and genotypes most likely result from the strong influence of transitory drought on the behavior of the studied barley accessions.

When interpreting the phenotypic reaction of each mutant and its reference genotype to the stress factor (understood as the difference between trait value in drought vs. control conditions) it can be indicated that none of the hormonal disorders influenced on the change of the number of internodes of main and lateral stems. This trait was independent of the genetic background and does not influence the plant architecture.

Our results demonstrated that impaired GAs biosynthesis and BRs signaling did not affect the behavior of the *sdw1* NILs and the *uzu1.a* mutant, respectively, in terms of length of the peduncle, length of the bottom and of internal internodes of the lateral stem. Despite the marked distance between the molecular response of Bowman and *uzu1.a* (BR) to late drought, their stress-induced phenotypic reaction was, among the studied NILs, the most similar to each other; this resulted also from the similar behavior of most of spike-related traits and length of the main stem. Similarly, the reaction of length and grain weight of the main spike were not influenced by GAs disorders in both *sdw1* NILs (compared to the Bowman reference genotype). In turn, largely distant molecular response of Sebastian and *hvdwarf14.d* (SL) was also manifested at the phenotypic level after transitory drought. Disturbed SLs signaling did not affect the reaction of only a few traits, including number of productive tillers, bottom internode length of main and lateral stems. This confirms the important role of SLs in plant development under stress conditions. Apparently, the SL signaling disorders caused profound modifications in *hvdwarf14.d* that could not be compensated during re-watering, which requires further investigation.

A large-scale transcriptomic study revealed numerous stress-induced changes in gene expression in mutants that were not found in the reference genotypes, and *vice versa*. Therefore, it can be assumed that disorders in the hormone pathway caused by the mutation is genes *HvGA20ox2*, *HvBRI1*, and *HvD14* strongly induced transcriptome remodeling in crowns under drought, although these genes, mutated or not, were not drought-responsive. Meanwhile, we identified common DEGs across the analyzed genotypes that may underlie the universal stress response and thus constitute valuable targets for improving cereal resistance to environmental hazards. These included genes annotated as LEA proteins, the lipolytic enzyme GDSL lipase/esterase, the DnaJ domain, and carbohydrate metabolism. Generally, they are known to play a critical role in plant response to drought.^58,59^ Carbohydrate remobilization is related to translocation of nonstructural carbohydrate components from non-leaf organs (such as crowns) to reproductive structures.^60^ In the case of cereal crops, the increased remobilization of carbon reserves is commonly regarded as a high-yielding mechanism in response to soil drying.^61^

### 4.1. Response to drought specific to sdw1 mutants

The phenotypes of *sdw1* mutants with disturbances in GA biosynthesis differed from the Bowman phenotype to a greater extent than their global transcriptomic profiles under drought conditions. Despite a large overlap of transcriptomic response to stress between Bowman and *sdw1* NILs the considerable differences were observed in the expression of genes related to GA signaling and biosynthesis (excluding *HvGA20ox2*). This can also affect the behavior of other drought-responsive genes. One such example is the DEGs annotated to the AP2/ERF domain. The transcription factor family AP2/ERF regulates several regulatory processes, such as plant growth and development, protection systems, metabolism-responsive genes in the signaling pathways of ethylene, and biosynthesis pathways of phytohormones, including ethylene (ET), CK, GA, JA, and ABA.^62^ This unique reaction of the mentioned DEG in genotypes with GA disorders may impact substantially on plant growth under adverse conditions. Moreover, our targeted quantification of phytohormones showed that across analyzed genotypes, GA_53_ levels increased remarkably only in Bowman plants under drought conditions. GA_53_ undergoes a series of oxidation steps mediated by *GA20ox* to produce GA20, which is then converted into bioactive GA_1_. Band et al.^63^ concluded that variations in GA_53_ accumulation are essential for creating a GA_1_ distribution that underpins growth regulation. Importantly, *sdw1* mutants are characterized by dysfunction of *HvGA20ox2*, which may explain the impaired oxidation of GA_53_ under drought conditions.

Similarly, most drought-responsive DEGs associated with brassinosteroids in Bowman were also identified in *sdw1* NILs. However, our study identified DEG with reduced expression in *sdw1* NILs that putatively encode BKI1 (an inhibitor of receptor kinases), which negatively regulates the BRI1 receptor in the brassinosteroid signaling pathway.^64^ Thus, we speculated that defects connected to the GAs hormonal pathway in *sdw1* NILs might be compensated for by BRs by limiting the inhibitory effect of BKI1 on BRI1 receptor activity under drought conditions. Additionally, two BR-related DEGs corresponded to GRAS TFs, and both had reduced expression in response to late drought in *sdw1.d*, whereas one of them (7HG0642830) was downregulated in *sdw1.a*. GRAS transcription factors are involved in hormone signal responses and biotic and abiotic stress reactions because many hormone- and stress-related cis-regulatory elements have been identified in the promoter regions of GRAS genes.^65^

Interestingly, *sdw1* mutants had the most numerous drought-induced changes in the expression of SL-related genes (discussed below), suggesting an interaction between GA and SL under drought conditions. Primarily, these DEGs corresponded to SLs signaling and were most abundantly annotated to the ubiquitin-conjugating enzyme E2. The covalent attachment of ubiquitin to specific target proteins occurs mainly through stepwise enzymatic cascade reactions, and ubiquitin is attached to substrates through coordinated ubiquitin-conjugating enzymes.^66^ Ubiquitin-mediated control of protein stability is central to most aspects of plant hormone signaling, including hormone perception and regulation of hormone biosynthesis. It should be pointed out that a comprehensive study regarding the role of E2 enzymes in plants remains unexplored.^67^ In contrast, strigolactone profiling in the present study revealed that in *sdw1* mutants, drought did not affect phytohormones accumulation.

Although *sdw1* NILs were genetically related, the contrasting drought response of some proteins was found between them. For instance, ACC oxidase and calreticulin were downregulated and upregulated in *sdw1.a* and *sdw1.d*, respectively. ACC is converted to ethylene by ACC oxidase. Notably, under stressful conditions, plants can produce high levels of ACC, which subsequently increases ethylene concentrations (ET stress), leading to an inhibition of plant growth and development.^68^ In turn, calreticulin is involved in many cellular processes in plants, such as protein folding and calcium homeostasis, and its upregulation is considered a self-protection mechanism.^69^ Therefore, we conclude that the *sdw1.d* mutant may have some adaptation mechanisms to facilitate the survival of plants under unfavorable osmotic conditions. In turn, dehydrin 9 may determine the acquisition of water stress-tolerance interconnected with GA pathway, since this protein was unaffected in both *sdw1* NILs and upregulated in Bowman during drought.

### 4.2. Response to drought specific to the uzu1.A mutant

Among the near-isogenic lines of Bowman studied, *uzu1.a* (a BR-signaling mutant) showed the most distant transcriptomic reaction to late drought compared to the reference genotype. In Bowman, more numerous alternations in BR-signaling genes were observed than in *uzu1.a*, similarly as in TCP TFs encoding genes annotated to drought response and SLs signaling. Such unaffected genes in mutant may play a specific role in modulation of BRs signaling during drought. Proteomic profiling indicated that the reaction of dehydrins to drought in Bowman was more efficient than in *uzu1.a*, because most of them were upregulated exclusively in the reference genotype under stress conditions; however, it was not confirmed at transcriptomic level. Additionally, we found that HVA22-like protein, belonging to the LEA family, exclusively overaccumulated in Bowman. Apparently, BRs signaling disorders resulted in no reaction of this protein to drought in mutant. Importantly, most HVA22 promoter sequences contain a large number of drought response elements (MYB), defense and stress response elements, and hormone response elements, suggesting that HVA22s may respond to adversity stresses and interact with phytohormones.^70^

Meanwhile, one of *uzu1.a*-specific DAPs was the malic enzyme, an important enzyme in plant metabolism that participates in the process of coping with stressful factors by increasing water-use efficiency or improving photosynthesis.^71^ Other *uzu1.a*-specific DAPs include sHSP domain-containing protein and several other proteins involved in sugar metabolism. All these play important roles in biological cell adaptation and osmoprotection in drought responses, linking growth and development.^72^ It is noteworthy that the *uzu1.a* mutant exhibited an exclusive drought-induced reaction of some hormones; namely, it was the only genotype with a marked increase in SA in response to late drought. On the other hand, the *uzu1.a* (BR), similar to the *sdw1.d* (GA) line, exhibited reduced levels of cis-zeatin and JA. This may indicate an antagonistic relationship between SA and both cytokinins and JA in the crowns of the BRs mutants. Indeed, it has been reported that SA can negatively regulate cytokinin signaling, which may lead to the fine-tuning of the effects of cytokinins on plant defense.^73^ On the other hand, the phenotypic characteristics of *uzu1.a* showed that drought treatment had the greatest negative influence (across genotypes) on spike-related-traits. Hence, it can be concluded that drought-induced overaccumulation of SA was insufficient to considerably improve the yielding of BR-signaling mutant.

### 4.3. Response to drought specific to the sdw1.D/uzu1.A mutant

GO enrichment analysis revealed that the drought response of the double mutant (GA/BR) was more similar to that of *uzu1.a* (BR) than *sdw1.d* (GA), especially during early drought; that is, the impairment effects of the BRs were larger than those of GAs. Our research showed that *uzu1.a* differed significantly from *sdw1* NILs, which may confirm the impact of BRs disorders on the GAs pathway; that is, BRs could affect GA biosynthesis by positively regulating *GA20ox* expression.^74^ GO term “polysaccharide catabolic process” was exclusively overrepresented in the double mutant, suggesting that reduced ability of cells to expand the polysaccharide network during water deficit, which limits the growth of plants by the reduced cell division in the meristematic zones.^75^

Interestingly, genes involved in the HSFA7/HSFA6b-regulatory network induced by drought and ABA initially increased under drought stress in the *sdw1.d/uzu1.a* mutant and then decreased during prolonged stress. Meanwhile, the ABA content increased in the double mutants over time. Based on a study of *HSFA6b*-knockout *A. thaliana* mutants, Huang et al.,^76^ reported that HSFA6b, which belongs to a class of heat shock factors (HSFs), is a positive regulator of ABA-mediated salt and drought resistance. Enhanced ABA accumulation is a hallmark of the plant response to drought, which in turn controls stomatal closure to decrease transpiration under drought.^77^

One DEG related to SLs biosynthesis (3HG0288380) exhibited a unique response to *sdw1.d/uzu1.a*. (the only gene from the analyzed set of genotypes with reduced expression), and was associated with carotenoid oxygenase. Carotenoids are important precursors of a large number of apocarotenoids and other compounds such as ABAs or SLs, and carotenoid oxygenases are key enzymes that degrade carotenoids.^78^ We also noted that DAPs, which were unaffected in the *sdw1.d* (GA) and *uzu1.a* (BR) mutants, were exclusively upregulated in the double mutant, including aquaporin, methionine S-methyltransferase, and chalcone-flavonone isomerase. Methionine S-methyltransferases are involved in many essential cellular processes, including biosynthesis, signal transduction, protein repair, chromatin regulation, and gene silencing. Chalcone-flavonone isomerase is a crucial rate-limiting enzyme in the flavonoid biosynthetic pathway; flavonoids are involved in the plant response to and protection from abiotic and biotic stresses.^79^ Hence, it can be claimed that upregulation of DEG or DAPs in *sdw1.d/uzu1.a* may provide additional survival mechanisms under adverse environmental conditions. Notably, only simultaneous disorders of growth-controlling hormones, namely GAs and BRs, in the double mutants contributed to the lack of negative effects of drought on plant height. Moreover, most spike-related traits and grain weight per plant were unaffected by stress in *sdw1.d/uzu1.a*, which makes this mutant a promising resource for improving drought tolerance in barley.

### 4.4. Response to drought specific to the hvdwarf14.D mutant

Any indication of the behavior of SLs signal transduction pathways is of great importance because SLs have not been sufficiently investigated, especially in barley.

Based on the present study, we demonstrated that the drought-induced transcriptomic reactions of the *hvdwarf14.d* (SL) mutant and Sebastian were substantially different, especially during late drought. These genotypes differed considerably in their response to drought in genes related to GAs, BRs, and SLs signaling. Generally, a much larger number of DEGs corresponding to the studied phytohormones were identified in Sebastian compared to *hvdwarf14.d*; therefore, it can be assumed that the mutation in *HvD14* provoked massive changes in numerous genes dependent on SL signaling involved in plant functioning under drought, not only those related to hormones. In contrast, the *hvdwarf14.d* mutant exhibited the largest number of DEGs, which were downregulated under early drought conditions and showed increased expression during prolonged stress. Some of these genes involved in hormone (ABA, SA, and JA) signaling or biosynthesis pathways. Therefore, it can also be assumed that perturbations in SLs metabolism may affect the functionality of other hormones in early drought, because these DEGs were not observed in Sebastian under early stress. Hormone profiling confirmed the different reactions in Sebastian and *hvdwarf14.d*. in relation to the GAs, BRs, and CKs. Meanwhile, the fold-change in ABA increase under drought conditions relative to the control was similar in these two genotypes. This is in accordance with the study by Marzec et al.,^30^ who did not observe drought-induced changes in ABA accumulation in the leaves of the barley *hvdwarf14.d* mutant compared to the Sebastian reference genotype; however, they found differences in the expression of genes involved in ABA biosynthesis. We also identified different reactions of some ABA biosynthesis and signaling genes in SL-signaling mutants exposed to drought. This may confirm the conclusion of Marzec et al.^30^ that disorders in ABA metabolism and signaling pathways may determine the specific behavior of SL-signaling mutants under drought conditions, since an interplay between SLs and ABA exists. Overall, the functional interpretation of the detected DEGs showed that most of the overrepresented GO terms in *hvdwarf14.d* during early drought were not enriched until late drought in its reference genotype. This finding suggests that the early reaction of the mutant corresponded mostly to the late reaction of Sebastian under drought. Four GO terms related to phosphorylation were enriched exclusively in the cv. Sebastian, mostly during early stress. This indicates that the defense mechanisms against stress in cv. Sebastian intensified via a phosphorylation mechanism in the subsequent days of stress more effectively than in the other genotypes. Intriguingly, drought-induced changes in expression of genes encoding kinases were less abundant in *hvdwarf14.d* (SL) than in Sebastian. This confirms the prominent function of strigolactones in phosphorylation-dependent signal transduction, because cascades of protein phosphorylation and dephosphorylation, mediated by kinases and phosphatases, influence gene expression, which plays an important role in signalosome affecting plant growth under abiotic stresses (Ma et al., 2021).^80^ Indeed, the phenotypic reaction to drought was substantially different between SL-mutant and the wild type.

Similar to transcriptomics, the proteomic profiles of *hvdwarf14.d* (SL) and its reference genotype Sebastian were substantially dissimilar over the course of stress and in various stress response pathways, including proline and tryptophan biosynthesis, known players conferring tolerance to abiotic stress.^81,82^ The downregulation of components of these pathways in the *hvdwarf14.d* mutant may have a negative impact on its phenotype under drought, such as the marked reduction in a thousand grain weight and number of tillers per plant. Evidently, the increased biosynthesis of defense proteins such as HSPs, and rubrerythrin at the onset of stress in SL-mutant was insufficient to overcame the negative effects of drought. We also noted that under drought the SLs signaling disorders may affect Water Stress and Hypersensitive response (WHy) domain-containing proteins which were overaccumulated only in Sebastian (not in *hvdwarf14.d*). Most likely, they belong to the LEA family and are involved in either the response to desiccation or bacterial infection.^83^

### 4.5. Co-reaction network and phytohormones crosstalk

The present study uncovered candidate genes for linkers in phytohormones crosstalk. Three of the bHLH-related DEGs affected by drought, corresponding to the signaling of both gibberellins and brassinosteroids, were found in *sdw1* NILs (GA disorders) and *sdw1.d/uzu1.a* (GA/BR). The bHLH transcription factor family is one of the largest transcription factor gene families important for plant growth and survival in adverse environmental conditions and is involved in the crosstalk of hormone signaling, including ABA, JA, BR, and SA.^84^ Some reports have suggested that bHLH/HLH proteins participate in BR hormone signaling pathways to promote cell elongation.^85^ Both BZR1 and BZR2, the key effectors of BR action, interact with the family of bHLH factors and GA signaling DELLA proteins to co-regulate the expression of a large number of genes, cell elongation, and photomorphogenesis.^86^ Our research suggests that the candidate linkers for the crosstalk between GAs and BRs belong to the bHLH transcription factor family.

Moreover, there were two late drought-responsive DEGs involved in GA and BR signaling: 2HG0153830 in *sdw1.d* and *sdw1.d/uzu1.a* and 2HG0153800 in Sebastian. Both genes were overexpressed under late drought and were annotated to aldo – keto reductase (AKR). AKR genes play a role in promoting stress resistance in plants by detoxifying reactive aldehydes and many redox reactions.^87^ Bartels et al.^88^ found that AKR4C1 protein in barley has an osmoprotective function during barley embryo development. In addition,^89^ observed that *AtAKR4C9* overexpression improves plant tolerance to salt stress.

Subsequently, we found a few drought-induced genes annotated to the WRKY domain, including WRKY50 and WRKY71, which are involved in BRs and JAs, as well as GAs and JAs signaling, respectively. WRKY transcription factors coordinate numerous plant functional processes, including responses to environmental stress.^90^ Genes encoding WRKY TFs identified in the present study may be strong candidates for drought improvement and hormone interplay mediators, including SA- and ABA-mediated signal pathways^91^ or negative regulators of the gibberellic acid-mediated signaling pathway via interaction between WRKY and DELLA proteins.^92^

Our co-reaction network construction, based on interactions between genes (related to GA, BR, SL, and drought), proteins, and hormones affected by drought revealed connections between DEGs and DAPs, mainly for GA-related genes. One of these DAPs (F2EF48) is HSP20. This finding indicates a possible tight interaction between HSP20 and GA-related genes in fluctuating environments that affect the growth, yield, and quality of barley. In fact, it has been suggested that most *HSP20s* are not expressed under normal conditions and respond to hormonal stimuli and abiotic stresses. The *cis-*elements of phytohormone-, light-, stress-responsive, and development-related genes have been identified in the *Hsp20* gene promoter sequences.^93^ Three GA-related DEGs were correlated with thaumatin activity under drought stress. Thaumatin-like proteins (TLPs) belong to the pathogenesis-related-5 (PR-5) family, are involved in stress responses, and play important roles in the regulation of plant growth.^94^ Therefore, we assume that growth regulation in crowns exposed to drought occurs via the interaction of TLPs with gibberellin-associated genes.

In turn, DEGs related to BRs were associated with several enzymes, including glutamine amidotransferase type-2 domain-containing protein, which, as described by Zhu and Kranz,^95^ is involved in the control of branching in *Arabidopsis*. One of the most important shot branching regulating factors is strigolactone; however, Zhu and Kranz^95^ showed that glutamine amidotransferase was not involved in SL biosynthesis since exogenously applied GR24 (a synthetic SL) does not correct the mutant phenotype. Thus, we suspected that the regulation of branching by glutamine amidotransferase in the crown, where the growth point is located, could be mediated by BR-related genes. Co-reaction network analysis also revealed an association between SL-related genes and peroxidase activity under water scarcity, which may confirm the participation of SL in determining the reaction to oxidative stress. Interestingly, this study also showed an interaction between glucose-6-phosphate 1-epimerase and JA, suggesting that JA may affect the metabolism of glucose-6-phosphatase, a substrate in glycolysis. Feng et al.^96^ documented that barley genes encoding glucose-6-phosphate dehydrogenases and other proteins responded to JA treatment, implying that JA may be a key regulator of *HvG6PDHs*. G6PDH plays a pivotal role in seed germination, nitrogen assimilation, plant branching, and plant responses to abiotic stress.^97^ Finally, we found remarkable interactions between the different types of hormones affected by drought, including the interplay between GA_20_ and IAA. The findings of Wolbang and Ross^98^ in peas indicated that an important function of apically derived auxin is to maintain normal GA biosynthesis in the elongating stems because IAA application dramatically increased the biosynthesis of the active GA, namely GA_1_, from its immediate precursor, GA_20_. In turn, an alternative metabolic pathway involving GA_20_ via inactive GA_29_ is inhibited by IAA. The second most significant interaction affected by drought was between strigol and kinetin. In rice, this interaction was revealed to regulate tiller development,^99^ and a similar mechanism has been proposed for barley crowns; however, this requires further investigation.

## 5. Conclusion

The multidisciplinary results expand our knowledge of barley crown performance under drought conditions. Examination of barley phytohormone mutants and reference genotypes proved that disorders in the functioning of gibberellins, brassinosteroids, and strigolactones resulting from the mutations in genes *HvGA20ox2*, *HvBRI1* and *HvD14*, respectively, substantially impacted the remodeling of the transcriptome, proteome, and hormones in response to drought; however, some effects of residual genetic polymorphisms cannot be excluded. This was also manifested at the phenotypic level, with some traits related to the main spike reacting to drought independent of the genetic background. We proved that strigolactones signaling disorder caused more profound multilevel alternations under drought than disturbances of other phytohormones. Components involved in the response to drought, which may be interconnected with phytohormones action, were identified by comparison of drought effects between mutant and reference genotype. Notably, the impaired biosynthesis of GAs affected the most and least the expression of SLs-related genes and BRs-related genes under drought, respectively. In turn, disturbance in BRs-signaling caused the most numerous changes in the expression of GAs-related genes in response to drought, and it had limited influence on regulation of BRs- and SLs-related genes in comparison with the reference genotype with normal BRs signaling. Meanwhile, SLs-signaling disorder affected primarily the expression of BRs-related genes under drought, whereas the number of GAs- and SLs-related DEGs under stress was greater in the reference genotype with normal SLs-signaling pathway. These findings indicate the genetic connections that may constitute the basis of interaction between all mentioned phytohormones in response to drought. Candidates that may underlie the genotype-universal stress response were detected, namely, genes annotated as LEA proteins, the lipolytic enzyme GDSL lipase/esterase, the DnaJ domain, and carbohydrate metabolism. They may constitute valuable targets for improving the resistance of cereals to environmental hazards. The present study uncovered candidate genes for linkers of phytohormone interplay, including genes encoding bHLH70 and bHLH101 as well as WRKY50 and WRKY71 transcription factors, during the barley crown response to drought. Data integration showed that branching in the crown exposed to drought may be affected by the expression of brassinosteroid-related genes, affecting the behavior of enzymes such as glutamine amidotransferase. Additionally, the interaction between hormones, namely gibberellins and auxins, as well as strigolactones and cytokinins, may regulate tiller development in barley crowns during drought.

## Supplementary Material

Supplemental Material

## Data Availability

The authors confirm that the data supporting the findings of this study are available within the article and its supplementary materials are available in Figshare at https://figshare.com/s/0dfe5e7e8b95f4c87b19. Additionally, RNA-seq data used in this paper are available in the ArrayExpress repository, accession number E-MTAB-13358 (https://www.ebi.ac.uk/biostudies/arrayexpress/studies/E-MTAB-13358).
