# Supplementary material for: Drought-induced molecular changes in crown of various barley phytohormone mutants
Source: Plant Signal Behav. 2024 Jun 26;19(1):2371693. doi: 10.1080/15592324.2024.2371693 (PMC11210921; doi:10.1080/15592324.2024.2371693)
Supplement: Supplemental Material [file KPSB_A_2371693_SM2799.zip › Figure S1.pdf]

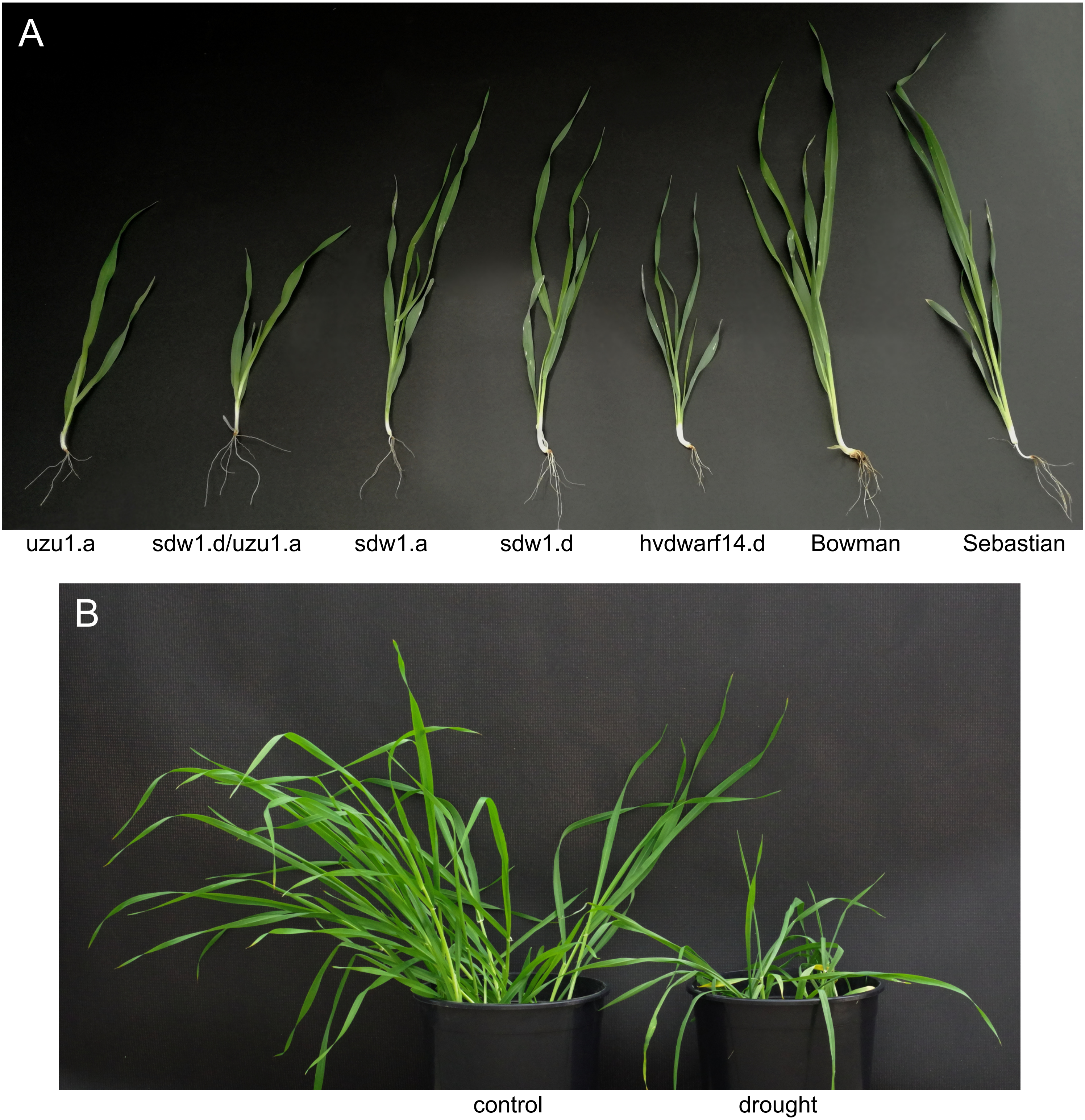

Figure S1. Barley phytohormone mutants and reference genotypes 20 days after sowing **(A)**; the Bowman reference genotype in control and drought conditions 34 days after sowing **(B)**
