# Supplementary material for: Drought-induced molecular changes in crown of various barley phytohormone mutants
Source: Plant Signal Behav. 2024 Jun 26;19(1):2371693. doi: 10.1080/15592324.2024.2371693 (PMC11210921; doi:10.1080/15592324.2024.2371693)
Supplement: Supplemental Material [file KPSB_A_2371693_SM2799.zip › Figure S2.pdf]

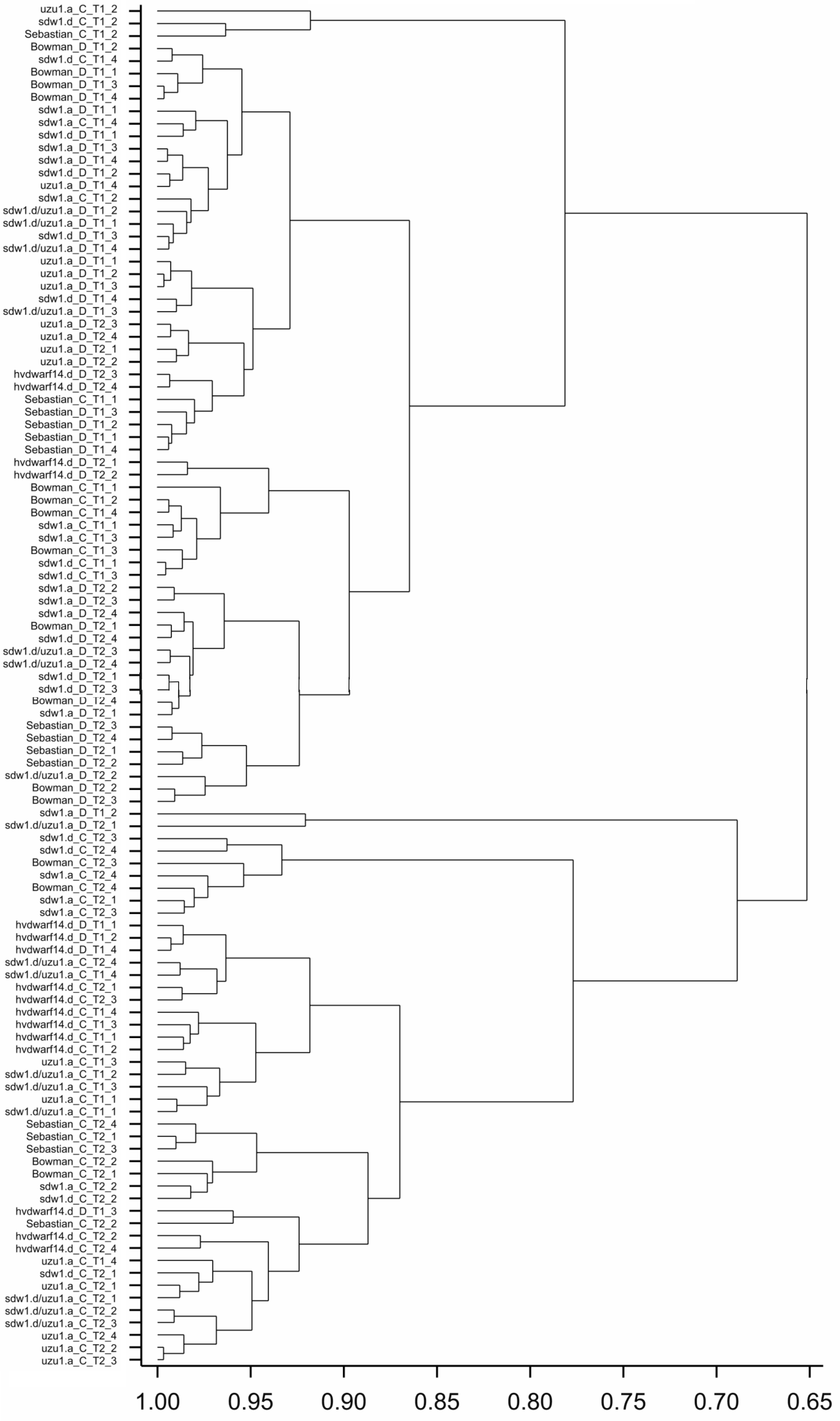

Figure S2A. Hierarchical clustering of 109 samples in mRNA-seq assay based on count data for 8,957 genes in the top quartile of between-samples variance; sample name from left to right as follows: genotype_treatment_time point_replication

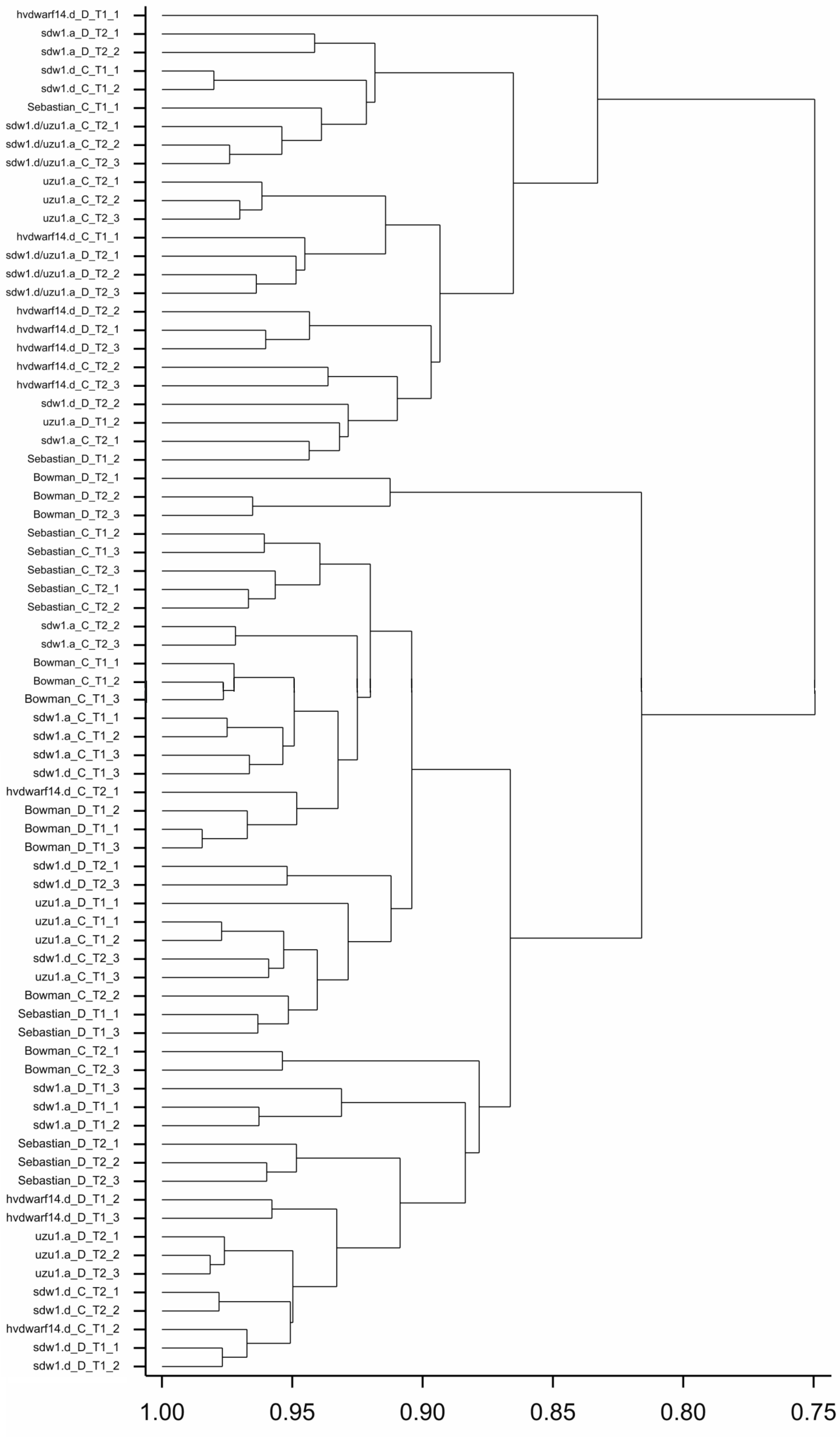

Figure S2B. Hierarchical clustering of 74 samples based on data for 4,970 proteins; sample name from left to right as follows: genotype_treatment_time point_replication
