# Supplementary material for: Drought-induced molecular changes in crown of various barley phytohormone mutants
Source: Plant Signal Behav. 2024 Jun 26;19(1):2371693. doi: 10.1080/15592324.2024.2371693 (PMC11210921; doi:10.1080/15592324.2024.2371693)

Figure S3. Clustering of genotypes based on various types of data. In (B-E) similarity coefficients based on euclidean distances computed from (drought - control) contrast estimates, complete link clustering algorithm

A. Kinship coefficients computed from 46170 SNP markers (only markers homozygous in all genotypes; average link clustering algorithm)

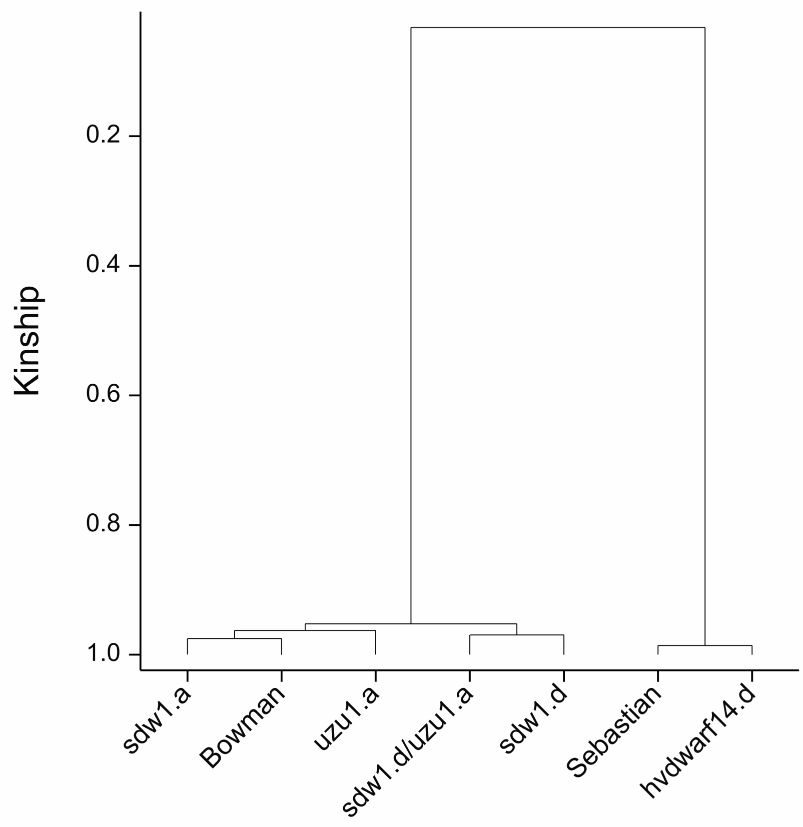

B. Clustering of genotypes based on differentially expressed genes in at least one comparison, at T1 and T2

T1

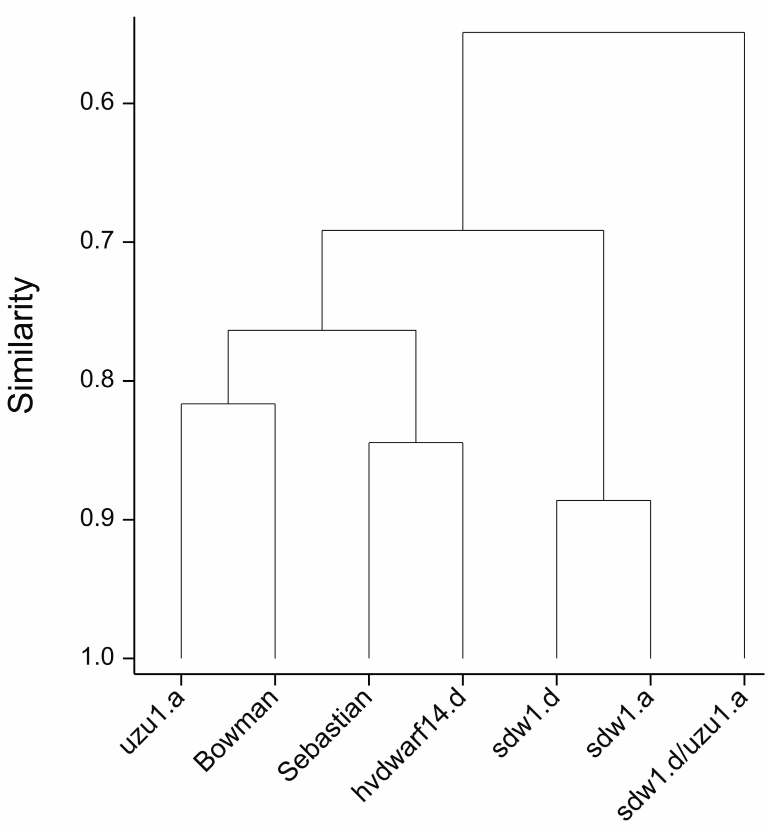

T2

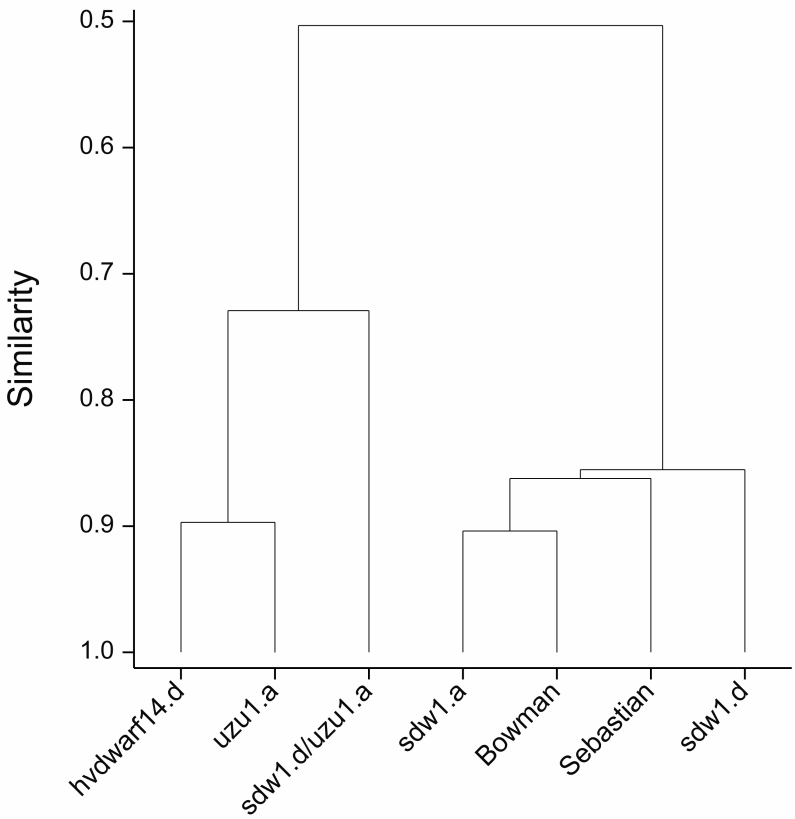

Figure S3. Continued

C. Clustering of genotypes based on differentially abundant proteins in at least one comparison, at T1 and T2

T1

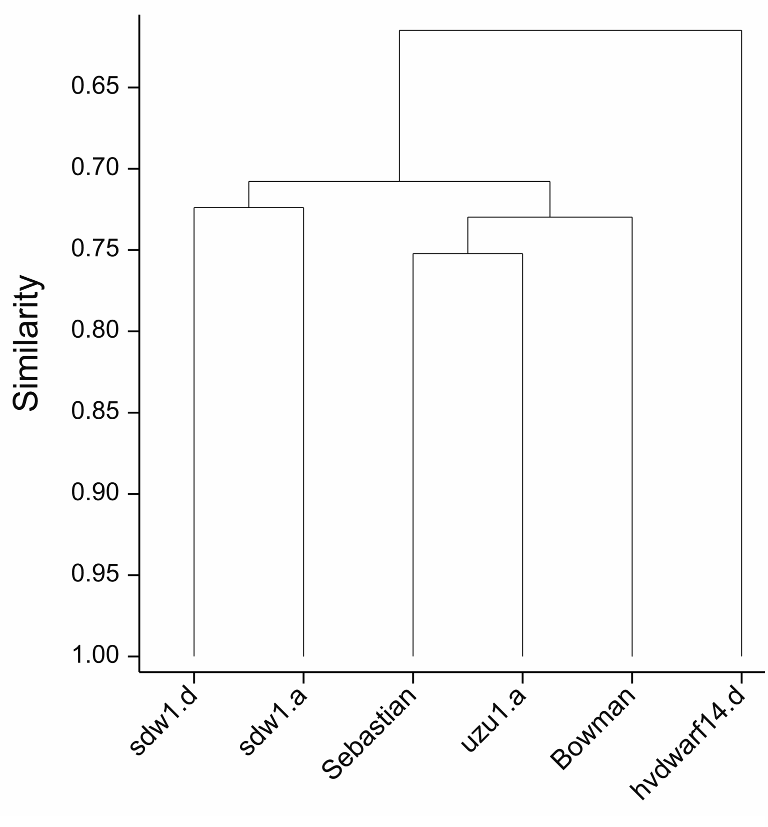

T2

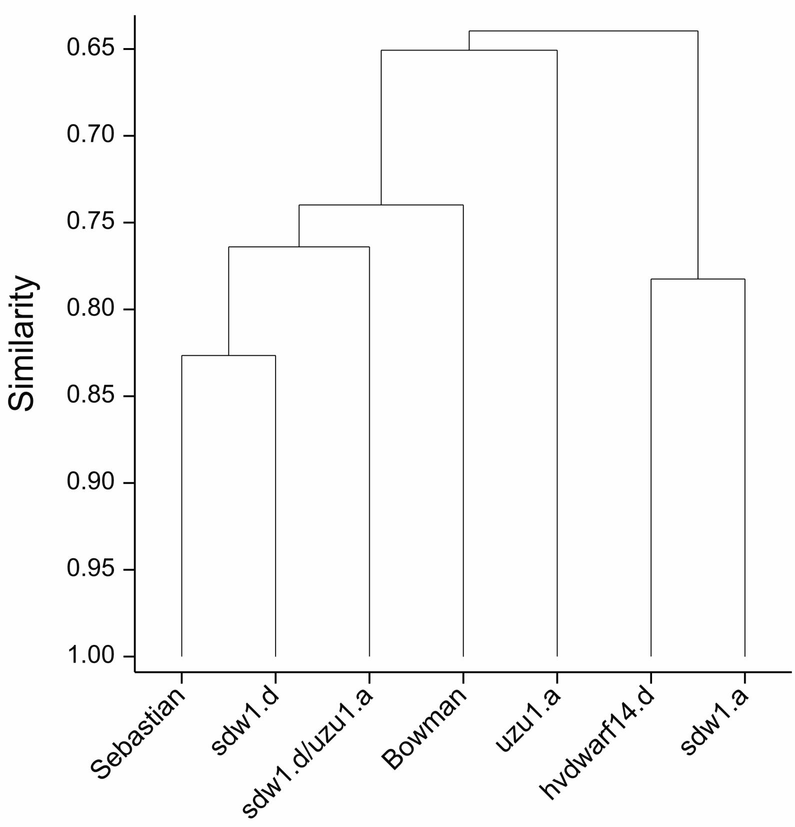

D. Clustering of genotypes based on phytohormones abundance at T1 and T2

T1

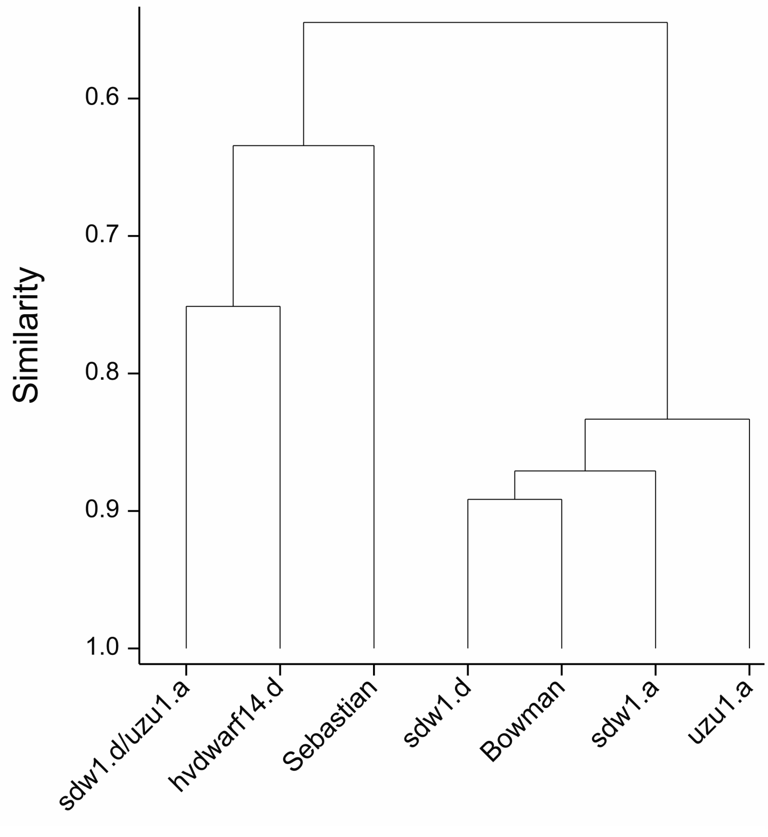

T2

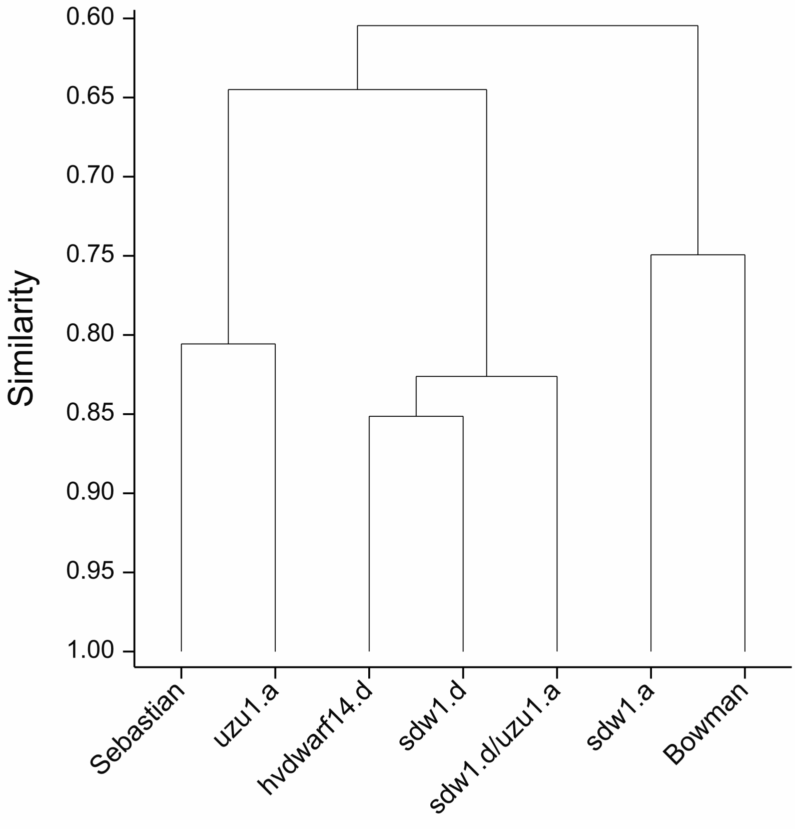

Figure S3. Continued

E. Clustering of genotypes based on post-harvest traits

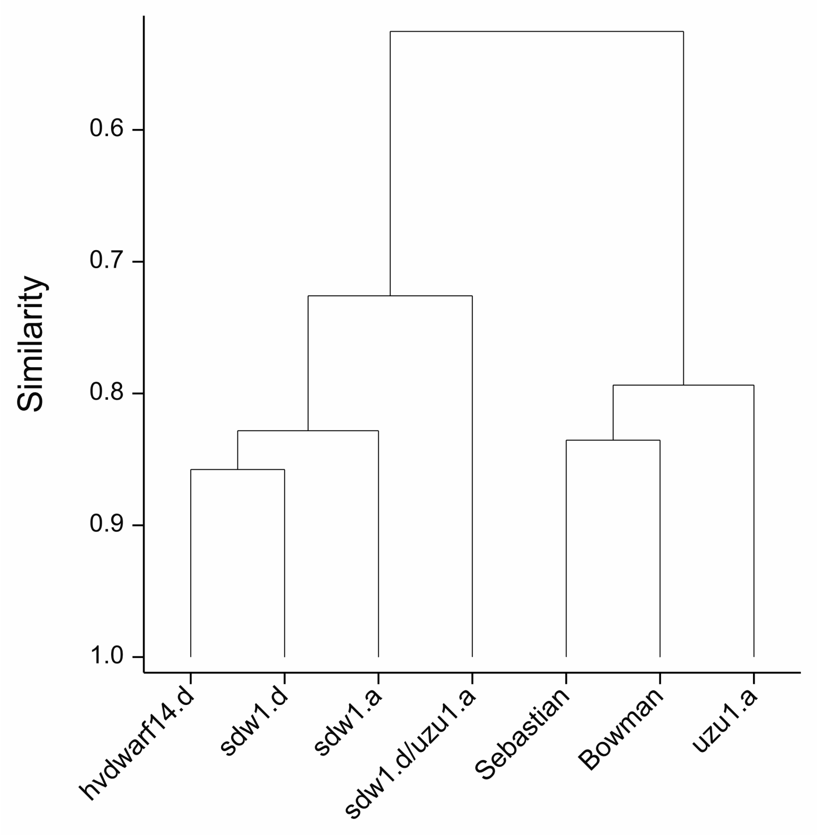

Supplement: Supplemental Material [file KPSB_A_2371693_SM2799.zip › Figure S3.pdf]
