# Supplementary material for: Drought-induced molecular changes in crown of various barley phytohormone mutants
Source: Plant Signal Behav. 2024 Jun 26;19(1):2371693. doi: 10.1080/15592324.2024.2371693 (PMC11210921; doi:10.1080/15592324.2024.2371693)
Supplement: Supplemental Material [file KPSB_A_2371693_SM2799.zip › Figure S4.pdf]

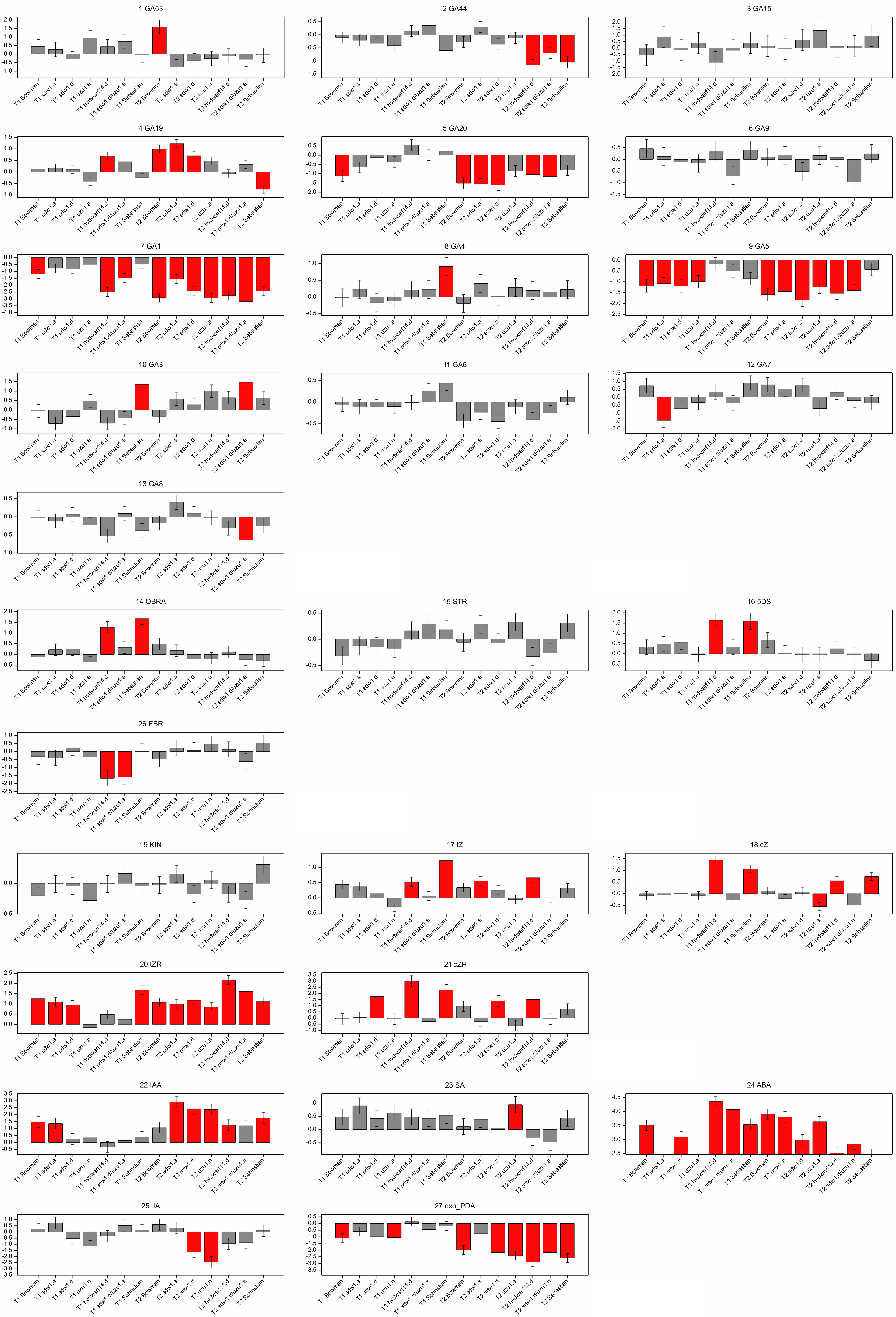

Figure S4. Contrasts between drought and control conditions calculated for analyzed phytohormones in seven barley genotypes in two time points (T1, T2); red - significant change, grey - not significant
